# Development and psychometric validation of a patient-reported outcome measure of recurrent urinary tract infection impact: the Recurrent UTI Impact Questionnaire

**DOI:** 10.1007/s11136-023-03348-7

**Published:** 2023-02-06

**Authors:** Abigail F. Newlands, Lindsey Roberts, Kayleigh Maxwell, Melissa Kramer, Jessica L. Price, Katherine A. Finlay

**Affiliations:** 1 School of Psychology and Clinical Language Sciences, University of Reading, Reading, RG6 7BE UK; 2 School of Psychology, University of Buckingham, Buckingham, MK18 1EG UK; 3 Department of Psychology, Faculty of Natural Sciences, University of Stirling, Stirling, FK9 4LA UK; 4 Live UTI Free Ltd, Ardeen House, 10-11 Marine Terrace, Dun Laoghaire, Dublin, Ireland

**Keywords:** Urinary tract infection, Recurrent urinary tract infection, Patient-reported outcomes, Patient-centred care, Women’s health, Chronic pain

## Abstract

**Purpose:**

Recurrent urinary tract infection (rUTI) is a highly prevalent condition associated with significant poor quality of life outcomes. A patient-reported outcome measure (PROM) of rUTI-associated psychosocial impact is urgently required to supplement clinical evaluation and validate the challenges experienced by patients. This study therefore developed and validated the Recurrent UTI Impact Questionnaire (RUTIIQ).

**Methods:**

A rigorous four-stage methodology was followed: (I) concept elicitation through a qualitative survey of the experiences of people with rUTI (*N* = 1983); (II) Delphi expert screening of the RUTIIQ with expert rUTI clinicians (*N* = 15); (III) one-to-one cognitive interviews with people experiencing rUTI (*N* = 28) to evaluate the comprehensiveness and comprehensibility of the RUTIIQ, and (IV) full pilot testing of the RUTIIQ with people experiencing rUTI (*N* = 240) to perform final item reduction and psychometric analysis.

**Results:**

Exploratory factor analysis demonstrated a five-factor structure comprising: ‘patient satisfaction’, ‘work and activity interference’, ‘social wellbeing’, ‘personal wellbeing’, and ‘sexual wellbeing’, collectively accounting for 73.8% of the total variance in pilot scores. Results from expert clinicians and patients indicated strong item content validity (I-CVI > .75). The internal consistency and test–retest reliability of the RUTIIQ subscales were excellent (Cronbach’s *α* = .81–.96, ICC = .66–.91), and construct validity was strong (Spearman’s *ρ* > .69).

**Conclusion:**

The RUTIIQ is a 30-item questionnaire with excellent psychometric properties, assessing the patient-reported psychosocial impact of living with rUTI symptoms and pain. This new instrument delivers the unique opportunity to enhance patient-centred care through standardised observation and monitoring of rUTI patient outcomes.

**Trial registration:**

This study was pre-registered with ClinicalTrials.gov (identifier: NCT05086900).

**Supplementary Information:**

The online version contains supplementary material available at 10.1007/s11136-023-03348-7.

## Plain English summary

Research has shown how recurrent urinary tract infections (rUTI) can severely impact quality of life, with negative consequences for mental health, socialising, the ability to engage with work and daily activities, and sexual wellbeing. Currently, there is no standardised approach for clinicians and researchers to evaluate this impact and understand how people are affected by living with rUTI. Therefore, in this study, a new validated questionnaire called a patient-reported outcome measure was developed. This questionnaire, called the Recurrent Urinary Tract Infection Impact Questionnaire, was developed with in-depth input from specialist clinicians in this field as well as a varied group of people living with rUTI. An initial test (or pilot) study of the questionnaire with a large group of people living with rUTI demonstrated its excellent statistical properties. This new tool provides the unique opportunity to enhance patient-centred care by supporting healthcare providers to understand the broad impact that this challenging condition may have, going beyond examination of symptoms alone.

## Introduction

Recurrent urinary tract infection (rUTI), characterised by at least two UTIs in six months or at least three in a year [1], affects over 100 million people annually worldwide and is associated with high UTI-related symptom and personal burden [2–4]. Qualitative research has emphasised the breadth of psychosocial challenges faced by people living with rUTI, indicating severe and long-lasting negative consequences for quality of life (QoL) [5–9]. Anxiety and depression are particularly common in this patient population [10], and are exacerbated by the high levels of sexual distress experienced by 60–78% of people with rUTI [11–14]. The societal implications of UTIs are also significant, with rUTI estimated to cost the NHS an increasing cost of £45 million per year in community-based prescriptions alone [14–16].

Patients regularly report symptoms of UTIs which are not indicated by standard clinical outcome measures [17, 18]. Exploring such patient-reported symptoms and associated impacts could offer a unique insight into the rUTI patient perspective and the lived experience of rUTI. Given the distinctive complexities faced by this patient cohort, it is surprising that no validated measure of rUTI impact on QoL currently exists. Generic QoL instruments, including EuroQol assessments such as the 5-item EQ-5D [19], are commonly used in UTI research [20, 21]. However, such instruments have not been specifically validated for use with rUTI and are thus not reliably sufficient to capture the rUTI patient experience. Their brief, generalised approach may not meaningfully reflect the spectrum of psychosocial challenges faced by people living with rUTI, and they may be less sensitive to changes in specific aspects of the rUTI experience [22]. Additionally, generic measures such as the EQ-5D do not explore the health-related impact on sexual wellbeing: an important area of impact for many people living with rUTI [8, 11, 14]. Indeed, recent research emphasises the need for clinicians and researchers to incorporate a condition-specific measure into rUTI care and study designs as standard, in combination with a brief generic measure such as the EQ-5D [22, 23].

A comprehensive, condition-specific patient-reported outcome measure (PROM) of rUTI impact on QoL would benefit clinical and research settings, supporting shared-decision making and patient-doctor interactions, highlighting key psychosocial outcomes that require intervention (for example, psychotherapy), and allowing for patient monitoring [22, 24, 25]. This study therefore aimed to develop and validate the Recurrent UTI Impact Questionnaire (RUTIIQ).

## Materials and methods

**Fig. 1:**
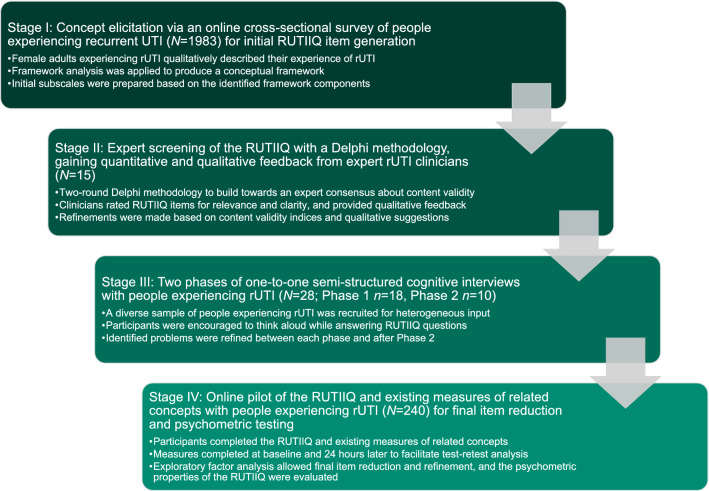
The four-stage methodology employed to develop and validate the Recurrent Urinary Tract Infection Impact Questionnaire (RUTIIQ), a novel patient-reported outcome measure of rUTI impact on quality of life. In-depth input from patient and expert clinician participants was maintained throughout this research, in line with best practice recommendations [26–28]

The RUTIIQ was developed and validated in accordance with PROM development guidelines (see Fig. 1) [26–28]. In-depth input from patients and expert clinicians was sought through all research stages [26–28], with particular emphasis on patient involvement to ensure the PROM is truly patient-centred [29].

### Stage I: concept elicitation

#### Design, setting, and participants

An online cross-sectional survey of adult females experiencing rUTI (*N* = 1983, see Table 1 for demographic characteristics) was conducted to collect qualitative data about the impact of living with this condition. Visitors to a website hosted by Live UTI Free (https://liveutifree.com), a UTI patient advocacy and research organisation, were invited to complete the survey. Inclusion criteria comprised a minimum age of 18 years old and meeting the diagnostic criteria for rUTI (≥ 2 UTI in 6 months, or ≥ 3 UTIs in 12 months) based on self-report [1, 18]. Informed e-consent was obtained prior to survey completion and participants could withdraw at any point.

**Table 1:** Participant demographic characteristics

Characteristic	*n*	%
Concept elicitation patients		
Country		
USA	1116	56.3
UK	383	19.3
Canada	105	5.30
Australia	90	4.54
India	33	1.66
Ireland	24	1.21
New Zealand	22	1.11
Germany	20	1.01
Other^a^	190	9.58
Total UTIs experienced		
2–3	185	9.33
4–6	340	17.2
7–10	395	19.9
11–15	297	15.0
> 15	766	38.6
Time between infections		
< 2 weeks	211	10.6
2–4 weeks	445	22.4
1–3 months	904	45.6
3–6 months	423	21.3
Typical symptom duration		
< 24 h	99	4.99
24–48 h	493	24.86
3–5 days	653	32.93
5–7 days	345	17.40
> 7 days	393	19.82
Expert clinicians		
Profession^b^		
General practitioner	8	53.3
Specialist doctor	6	40.0
Specialist nurse practitioner	1	6.67
Gender		
Female	12	80.0
Male	3	20.0
Country of practice		
United States	8	53.3
United Kingdom	6	40.0
Canada	1	6.67
Ethnicity		
White	10	66.7
Asian	4	26.7
Other	1	6.67
Cognitive interview patients		
Gender		
Female	25	89.3
Male	2	7.14
Non-binary	1	3.57
Country of residence		
United States	9	32.1
United Kingdom	7	25.0
Canada	4	14.3
Australia	2	7.14
Austria	1	3.57
Netherlands	1	3.57
New Zealand	1	3.57
South Africa	1	3.57
Ukraine	1	3.57
United Arab Emirates	1	3.57
Ethnicity		
White	25	89.3
Asian	2	7.14
Spanish or Latino American	1	3.57
Fluency in English		
Native or bilingual	25	89.3
Advanced or proficient	3	10.7
Relationship status		
Married or in a civil partnership	17	60.7
In a relationship	6	21.4
Single	4	14.3
Separated or divorced	1	3.57
Pilot patients		
Gender		
Female	233	97.1
Male	5	2.08
Non-binary	2	.83
Country of residence		
United States	93	38.8
United Kingdom	77	32.1
Australia	18	7.50
Canada	16	6.67
France	4	1.67
Sweden	4	1.67
Malaysia	3	1.25
New Zealand	3	1.25
Spain	3	1.25
Other^c^	19	4.58
Ethnicity		
White	214	89.2
Asian	11	4.58
Spanish or Latino American	6	2.50
Mixed	2	.83
Black or African American	1	.42
Native Hawaiian or other Pacific Islander	1	.42
Prefer not to say	5	2.08
Fluency in English		
Native or bilingual	195	81.2
Advanced or proficient	45	18.8
Relationship status		
Married or in a civil partnership	123	51.3
In a relationship	78	32.5
Single	32	13.3
Engaged	4	1.67
Widowed	1	.42
Prefer not to say	2	.83

Concept elicitation patients *N* = 1983. Expert clinicians *N* = 15. Cognitive interview patients *N* = 28 (*n* = 18 in Phase 1, *n* = 10 in Phase 2). Pilot patients *N* = 240 (*n* = 106 participants completed the Test–Retest Analysis survey 24 h later)

^a^Other countries of residence where *n* < 15

^b^Of the specialist clinicians, 71.4% (*n* = 5) worked in urology and 28.6% (*n* = 2) in urogynaecology

^c^Other countries of residence where *n* < 3 comprise: Austria, Costa Rica, Germany, Greece, India, Ireland, Kenya, Netherlands, the Philippines, Portugal, Romania, Saint Vincent and the Grenadines, South Africa, Switzerland, and Ukraine

Most participants were from the USA (56.3%, *n* = 1,116), UK (19.3%, *n* = 383), Canada (5.30%, *n* = 105), and Australia (4.54%, *n* = 90). Participant ages were broadly distributed across the sample, with 56.7% (*n* = 1,126) aged between 18 and 39 years old and 43.2% (*n* = 857) aged 40 years old or above. Most participants reported that their UTI episodes last between 1 and 5 days long (57.8%, *n* = 1,146) and that they have 1 to 3 months between infections (45.6%, *n* = 904). Over a third of participants had experienced more than 15 UTIs (38.6%, *n* = 766).

#### Procedure

After providing consent, participants answered questions about their demographic characteristics and clinical history with rUTI, including frequency and duration of symptoms. Next, participants were asked to qualitatively describe their experience of rUTI via an open-text box.

#### Data handling

Framework analysis of the qualitative data and a thorough literature review were conducted to produce a conceptual framework of rUTI impact (see Online Resource 1 for data handling strategy and see Online Resource 2 for summary of literature consulted) [28, 30, 31]. After data familiarisation, the first author developed an initial thematic framework of psychosocial components based on factors identified a priori from existing literature and emergent issues raised by participants [31]. The entire research team reviewed this collaboratively in conjunction with the dataset and literature review findings, making agreed revisions. The first author then indexed and annotated the full dataset according to this framework, and patterns were charted and mapped to group the data into defined concepts (see Online Resource 3 for thematic framework) [31]. Secondary coding and triangulation were undertaken by the last author. Both coders hold advanced postgraduate training in research methods and extensive qualitative experience (see Stage III). Disagreements were resolved in discussion with the wider research team and in close consultation with the data. Data saturation was achieved. Each overarching framework component was selected for exploration as a preliminary subscale in the RUTIIQ, and initial items were drafted for each according to the relevant framework subcomponents.

### Stage II: expert clinician screening

#### Design, setting, and participants

A Delphi methodology was employed to evaluate the content validity of the initial questionnaire from the perspective of clinicians with rUTI expertise (see Online Resource 4 for all items tested at this stage) [32]. Two rounds of anonymous surveys took place to build towards an expert consensus about the relevance and clarity of the questionnaire items [32].

Thirty-seven expert clinicians were invited to take part (*n* = 22 female, *n* = 15 male), of whom 15 were successfully recruited (*n* = 12 female, *n* = 3 male), meeting sample size recommendations (see Table 1 for demographic characteristics) [26, 32]. Purposive recruitment with snowball sampling was applied to obtain a heterogeneous sample with an equal proportion of clinicians working in primary and secondary care to reduce the risk of bias [33, 34]. Inclusion criteria comprised those currently working as either a general health practitioner or specialist doctor/nurse practitioner within urology or an allied discipline.

The expert clinicians were aged between 32 and 64 years old (*M* = 46.8, *SD* = 9.24) and their experience in treating rUTI ranged from 2 to 30 years (*M* = 13.2, *SD* = 7.95). Approximately half of the expert clinicians were general practitioners (53.3%, *n* = 8), and half were specialist doctors or nurse practitioners (46.7%, *n* = 7). Specialists practised within urology (71.4%, *n* = 5) and urogynaecology (28.6%, *n* = 2). The participants practised in the USA (53.3%, *n* = 8), UK (40.0%, *n* = 6), and Canada (6.67%, *n* = 1). Eighty percent retention was achieved in Round 2, supported by regular personalised email reminders.

#### Procedure

RUTIIQ items were presented using an online survey tool (REDCap; https://www.project-redcap.org). In Round 1, the clinicians were asked to rate each for relevance and clarity using a 7-point scale (0 = not at all relevant/clear, 6 = highly relevant/clear) and provide qualitative comments about comprehensiveness and comprehensibility [26, 32]. In Round 2, each RUTIIQ item was presented alongside the median relevance and clarity ratings and anonymised qualitative feedback from Round 1. The expert clinicians either retained or updated their original ratings and provided further qualitative feedback.

#### Data handling

Median ratings from Round 2 were calculated and analysed in conjunction with qualitative feedback. Content validity indices for items (I-CVI) were computed by dividing the number of experts who scored an item’s relevance/clarity as at least 4 out of 6 by the total number of experts [35]. A threshold of 0.75 was specified a priori as the minimum I-CVI required to indicate acceptable consensus of content validity, with a minimum median score of 4 [32, 34].

### Stage III: patient cognitive interviews

#### Design, setting, and participants

To evaluate how the RUTIIQ may be mentally processed and where problems may arise, one-to-one semi-structured interviews were conducted with rUTI patients using Microsoft Teams [36]. Cognitive debriefing techniques were used to encourage participants to think aloud as they answered the RUTIIQ questions (see Online Resource 5 for all items tested at this stage) [36]. Interviews took place in two phases, with interim iterative refinements validated in the second phase [37].

All cognitive interviews were conducted by the first author to ensure homogeneity in interview style and to facilitate rapport and participant comfort [28, 36]. The interviewer had advanced postgraduate training in conducting and analysing qualitative interviews, and ongoing training and quality monitoring were provided by the last author who has extensive expertise as a Chartered Health Psychologist and academic qualitative researcher. To facilitate transcription, all interviews were audio-recorded. Full ethical approval was granted by the School of Psychology and Clinical Language Sciences Research Ethics Committee, University of Reading (project reference: 2021-043-KF). Anonymised recordings were securely stored on the University of Reading server, only accessible to the research team. Data were retained for five years from the study’s completion and destroyed sooner if requested. APA guidelines for ethical conduct were maintained at all times [38].

A clinically and demographically diverse sample of 28 adults experiencing rUTI was purposively recruited (see Table 1 for demographic characteristics). To reduce risk of selection bias and sampling error, a large sample representative of the different subgroups that make up the rUTI patient cohort was sought via a broad recruitment strategy with two possible sources: (1) via people signed up to receive newsletters and research notifications from a key stakeholder group: Live UTI Free (https://liveutifree.com), and (2) via other UTI-related online sources, such as support groups. Interested participants were encouraged to share the study information on social media. Recruitment was incentivised using a £25 online shopping voucher prize draw for one random winner, aiming to reduce dropout and attrition bias [39].

Inclusion criteria comprised a minimum age of 18 years old, native or advanced fluency in English, and meeting the diagnostic criteria for rUTI based on self-report [1, 18]. Participants who reported a current diagnosis of interstitial cystitis, were using urinary catheterisation, or were pregnant were excluded. Seventy-three potential participants completed a screening survey without exclusion, from which as diverse a sample as possible was selected via maximum variation sampling (see Online Resource 6) [40]. A minimum sample size of 25 participants was sought to achieve confidence that all possible problems with the questionnaire had been identified [26, 41]. With a final sample of 28 interviewed participants (*n* = 18 in the first interview phase, and *n* = 10 in the second interview phase), sampling adequacy was reached.

The final sample had an age range of 18 to 82 years old (*M* = 46.8, *SD* = 16.9), and comprised 92.9% females (*n* = 26) and 7.14% males (*n* = 2), with one participant describing themselves as non-binary and assigned female at birth. Participants resided in 10 countries, predominantly the USA (32.1%, *n* = 9), the UK (25.0%, *n* = 7), and Canada (14.3%, *n* = 4). The median number of UTI episodes in the past 6 and 12 months was 4 (*IQR* = 4) and 7 (*IQR* = 8), respectively. Years of UTI symptoms ranged from 1 to 65 (*M* = 17.3, *SD* = 14.5) and years of UTI impact to QoL ranged from 1 to 60 (*M* = 8.90, *SD* = 12.0).

#### Procedure

Participants provided e-consent after reviewing the study’s ethical considerations. The interviewer presented the RUTIIQ to participants using a ‘screen-share’ function and invited them to think aloud their thought processes whilst deciding their answer for each question, allowing evaluation of the measure’s overall comprehensiveness and comprehensibility [26, 28, 37]. Informed by Willis’ guidance on planning and conducting cognitive interviews for instrument development [36, 37], a topic guide (Online Resource 7) was designed and employed to semi-structure each interview, including scripted probes to encourage elaboration and clarification [37]. Questions about the scale and response options, the time taken to answer questions, and the questionnaire layout and formatting were also asked [36]. Anonymised field notes and written summaries were prepared throughout and following each interview to build richness and support transcript interpretation [37].

#### Data handling

Anonymised verbatim interview transcripts of the audio recordings were created using speech-to-text transcription software (Otter; https://otter.ai/), with errors manually corrected. Predefined codes from the Question Appraisal System (QAS-99) [42], designed to support the systematic assessment of questionnaire items and identification of potential problems, were used to support question feature coding. The QAS-99 training manual was applied to create a coding system for the dataset, systematically evaluating every participant response to each RUTIIQ item in relation to question features (for example, comprehension of technical terms). Initial coding was conducted by the first author, with triangulation undertaken by the last author. This assessment was supported by verbatim participant quotes and interviewer field notes [37].

Weekly wider research team meetings were held, allowing for in-depth analysis of transcripts, drawing together a variety of perspectives from expertise including experience in academic mixed-methods research, clinical practice, and patient advocacy. If there were any uncertainties found in Phase 1, the decision was taken to retain the item in Phase 2 and gain further feedback. A third version of the RUTIIQ was created after Phase 1 for assessment in Phase 2, with the same process undertaken at the end of Phase 2 to create a fourth version for pilot testing.

### Stage IV: RUTIIQ pilot testing

#### Design, setting, and participants

To collect data for psychometric testing of the RUTIIQ and final item reduction, a two-part cross-sectional survey of adults experiencing rUTI was conducted online. Participants completed the same procedure twice to facilitate test–retest analysis [26–28]: (1) at baseline, and (2) 24 h later (Test–Retest Assessment). A maximum time period of 48 h to complete the Test–Retest Assessment was applied following the minimum satisfactory test–retest window advocated by Streiner, Norman & Cairney [43]. This prioritised within-episode stability and reduced the possibility of a separate rUTI episode altering reporting of rUTI symptom impact [44].

In addition to completing the RUTIIQ, participants completed existing validated measures relating to each RUTIIQ subscale to facilitate construct validity [27, 28]. It was hypothesised a priori that there would be moderate to strong correlations (Spearman’s *ρ* > 0.50) between the RUTIIQ subscale scores and existing instruments measuring constructs related to the RUTIIQ subscales (‘concurrent measures’) [27, 28].

A sample of 240 adults meeting the diagnostic criteria for rUTI completed the Baseline Assessment, of whom 106 (44.2%) completed the Test–Retest Assessment (see Table 1 for demographic characteristics, and Online Resource 8 for sampling and recruitment strategy). Participants were recruited using the same recruitment channels used in Stage III, also applying the same inclusion and exclusion criteria. At least 210 participants were required to complete the Baseline Assessment to perform final item reduction via exploratory factor analysis with at least 5 participants per questionnaire item (RUTIIQ = 42 items before final item reduction). Sampling adequacy was exceeded.

The final sample (*N* = 240) was aged between 18 and 84 years old (*M* = 45.0, *SD* = 17.3), and comprised 97.9% females (n = 235) and 2.08% males (*n* = 5), with two participants describing themselves as non-binary and assigned female at birth. Twenty-four countries were sampled, with most participants residing in the USA (38.8%, *n* = 93), the UK (32.1%, *n* = 77), Australia (7.50%, *n* = 18), and Canada (6.67%, *n* = 16).

Approximately half of the participants (56.3%, *n* = 135) reported taking antibiotics at the time of participation, either to treat a current UTI, prevent new UTIs, and/or for other indications. Approximately three-quarters (76.3%, *n* = 183) reported managing their rUTI with non-antibiotic treatment including natural remedies or supplements. More than three-quarters of participants (77.5%, *n* = 186) reported experiencing persistent lower urinary tract symptoms for at least the past three months, with the remainder reporting symptoms which occur on an episodic basis. The mean number of episodes of symptoms reported in the past six months was 6.81 (*SD* = 24.3), and the mean in the past year was 13.9 (*SD* = 48.4).

#### Procedure

In the Baseline Assessment, participants provided e-consent via REDCap after reviewing the study’s ethical considerations. Eligible participants who were not excluded during a screening questionnaire proceeded to complete the RUTIIQ and the following six concurrent measures: the Patient Health Questionnaire 9 (PHQ-9) to assess symptoms of depression [45], the Generalized Anxiety Disorder 7 (GAD-7) to assess levels of anxiety [46], the University of Los Angeles Loneliness Scale Version 3 to explore feelings of loneliness and social isolation [47], the Work Productivity and Activity Impairment Questionnaire for Specific Health Problems to evaluate the health-related impact on carrying out work and daily activities [48], the Female Sexual Distress Scale–Revised (FSDS-R) to measure sexual dysfunction [49], and the Patient Satisfaction Questionnaire 18 to assess patient-reported satisfaction with healthcare [50]. The Test–Retest Assessment included the same instruments.

#### Data handling

After preparing the data for analysis (see Online Resource 9 for data handling strategy [51–60]), summed scores were calculated for each RUTIIQ subscale and each concurrent measure. Exploratory factor analysis was conducted to perform final item reduction and determine the latent factor structure (structural validity) of the RUTIIQ [26, 28, 53]. Further psychometric analyses of the RUTIIQ, comprising test–retest reliability, internal consistency, and construct validity, were conducted and compared to gold-standard recommendations [27, 28]. Linear regression analyses were also performed to examine any measurement invariance in RUTIIQ scores (for example, related to sociodemographic differences). The Automated Readability Index, a readability measure known to be especially applicable to non-narrative text such as questionnaires [58], was computed to estimate the literacy level required for comprehension of the RUTIIQ.

## Results

### Stage I: concept elicitation

Framework analysis of the participants’ qualitative responses resulted in a conceptual framework comprising five key components: *personal wellbeing*, *social wellbeing*, *work and activity interference*, *sexual wellbeing*, and *patient satisfaction* (see Online Resource 3 for thematic framework and supporting verbatim quotations). These were selected for exploration as five subscales in the RUTIIQ, with initial items based on their subcomponents.

### Stage II: expert clinician screening

All items achieved I-CVI for relevance and clarity greater than 0.75 and median ratings of at least 4 as specified a priori. Minimal item refinements were implemented based on qualitative recommendations to enhance clarity, by providing more detailed instructions and definitions (see Online Resource 4 for refinements and I-CVI).

### Stage III: patient cognitive interviews

Qualitative feedback from patient participants in the cognitive interviews suggested that clarity and comprehensibility could be improved by giving examples and simplifying language (see Online Resource 5 for refinements and verbatim quotations). Refinements made after Phase 1 were tested during Phase 2, after which only minor changes were made with no new items added; data saturation was therefore reached [37].

### Stage IV: RUTIIQ pilot testing

#### Exploratory factor analysis

Bartlett’s Test of Sphericity was statistically significant (*p* < 0.001), indicating the absence of multicollinearity [53]. The Kaiser–Meyer–Olkin Measure of Sampling Adequacy estimate was high at 0.89, confirming the suitability of the data for exploratory factor analysis [53]. All extracted communalities were greater than 0.40 except for item A5 (impact on diet), which also did not load above 0.40 on any initial factors and was therefore removed. All other items met these thresholds for communalities and factor loadings. Items demonstrating multiple cross-loadings (B1: impaired close relationships; B3: impaired social activities; B7: worrying about being a burden to others) were removed.

The final five-factor structure comprises the following factors: ‘patient satisfaction’, ‘work and activity interference’, ‘social wellbeing’, ‘personal wellbeing’, and ‘sexual wellbeing’ (see Table 2), together accounting for 73.8% of the total variance in scores. They represent a strong fit for the data, clearly distinguishing between the RUTIIQ subscales. The final version of the RUTIIQ consists of 30 items in total (see Table 3; the full questionnaire is available in Online Resource 10).

**Table 2:** Final five-factor structure of the RUTIIQ

Factor: item	Factor loading					Communality
	1	2	3	4	5	
Factor-1: Patient satisfaction^a^						
E1. I have generally felt content with the medical care I have been receiving	**.83**	− .07	.09	− .06	− .09	.71
E2. I have felt confident about being able to get the medical care I need	**.87**	− .07	.07	− .06	− .13	.79
E3. I have felt like my medical concerns are taken seriously	**.88**	− .06	− .09	− .08	− .03	.80
E4. I have had confidence in the decisions made about my care and treatment	**.88**	− .07	.05	− .08	− .03	.80
E5. I have felt confident about being able to access UTI testing and treatment quickly enough	**.68**	− .05	.00	− .04	.00	.47
E6. I have felt listened to by my healthcare provider(s)	**.89**	− .09	− .07	− .13	− .02	.82
E7. I have had easy access to the medical specialists I need	**.79**	− .03	.04	.04	− .04	.64
E8. I have felt like my healthcare provider(s) treat me with respect and dignity	**.82**	− .06	− .23	− .06	.00	.73
E9. I have been as involved as I have wanted to be in the decisions made about my care and treatment	**.72**	− .08	− .06	− .03	.01	.52
E10. I have trusted my healthcare provider(s)	**.89**	− .11	− .06	− .06	− .02	.81
Factor-2: Work and activity interference						
C1. I regularly missed full or partial days of work, home responsibilities or studying	− .08	**.79**	.21	.09	.06	.69
C2. My ability to work was impaired	− .06	**.91**	.18	.13	.04	.87
C3. I regularly put pressure on myself to work despite feeling unwell	− .08	**.61**	.22	.18	.23	.51
C4. The kind or amount of work I could do was limited	− .10	**.85**	.25	.09	.02	.80
C5. It was more difficult than usual to concentrate on my work	− .14	**.78**	.25	.29	.10	.78
C6. It was more difficult than usual to handle my workload	− .11	**.84**	.26	.22	.08	.84
C7. I have felt that the quality of my work was lower than usual	− .12	**.83**	.20	.16	.10	.78
Factor-3: Social wellbeing						
B1. I have felt alone or isolated from others	− .10	.37	**.68**	.29	.15	.72
B2. I have avoided socialising more than I used to	.04	**.47**	**.66**	.20	.17	.73
B3. I have felt embarrassed in social situations	− .07	.33	**.75**	.13	.05	.70
B4. I have felt that I am no longer close to anyone	− .01	.30	**.63**	.22	.12	.55
B5. I have felt anxious in social situations	.03	.35	**.70**	.26	.11	.69
Factor-4: Personal wellbeing						
A1. I have experienced feelings of anxiety	− .12	.20	.19	**.68**	.20	.60
A2. I have experienced feelings of low mood or depression	− .10	.29	.24	**.88**	.16	.95
A3. I have felt hopeless about the future	− .16	.26	.29	**.72**	.16	.71
A4. I have had poor or disrupted sleep	− .06	.33	.26	**.46**	.12	.40
Factor-5: Sexual wellbeing						
D1. I have avoided sexual activity to minimise the risk of developing or worsening UTI symptoms	− .07	.06	.12	.12	**.69**	.52
D2. I have felt unable to enjoy sexual activity due to my UTI(s)	− .05	.20	.09	.15	**.63**	.47
D3. I have been concerned about the impact of my UTI(s) on my sex life and/or sexual relationship(s)	− .05	.07	.02	.07	**.79**	.64
D4. I have felt that my UTI(s) have made my sexual wellbeing worse	.00	.03	.10	.09	**.76**	.60

*N* = 183. A five-factor structure was identified, indicating the distinctive areas of impact associated with recurrent urinary tract infections. These five factors together accounted for 73.8% of the total variance in scores. The extraction method was Principal Axis Factoring with Kaiser-Varimax rotation. Factor loadings above .40 are in bold [53, 55]

^a^Patient satisfaction subscale scores were reverse scored

**Table 3:** Final 30 items included in the RUTIIQ

Section/item number	Instruction/item
Section A: Personal wellbeing	The following questions are about the impact of your UTI(s) on your personal wellbeing Thinking about how you have felt in the past TWO WEEKS, please indicate how strongly you agree or disagree with the following statements: Because of my UTI(s)…
A1	I have experienced feelings of anxiety
A2	I have experienced feelings of low mood or depression
A3	I have felt hopeless about the future
A4	I have had poor or disrupted sleep
Section B: Social wellbeing	The following questions are about the social impact of your UTI(s) Thinking about how you have felt in the past TWO WEEKS, please indicate how strongly you agree or disagree with the following statements: Because of my UTI(s)…
B1	I have felt alone or isolated from others
B2	I have avoided socialising more than I used to
B3	I have felt embarrassed in social situations
B4	I have felt that I am no longer close to anyone
B5	I have felt anxious in social situations
Section C: Work and activity interference	The following questions are about the impact of your UTI(s) on your work and/or regular daily activities. Please consider the term “work” to include paid employment, volunteering, home management, caring responsibilities, and/or studying Thinking about how you have felt in the past TWO WEEKS, please indicate how strongly you agree or disagree with the following statements: Because of my UTI(s)…
C1	I regularly missed full or partial days of work, home responsibilities or studying
C2	My ability to work was impaired
C3	I regularly put pressure on myself to work despite feeling unwell
C4	The kind or amount of work I could do was limited
C5	It was more difficult than usual to concentrate on my work
C6	I have felt that the quality of my work was lower than usual
C7	It was more difficult than usual to handle my workload
Section D: Sexual wellbeing^a^	The following questions are about the sexual impact of your UTI(s)
	Do you feel your UTI(s) has/have impacted your sex life in the past two weeks?
	If you selected “No” or “Prefer not to say”, please skip to Section E. If you selected “Yes”, please continue with the rest of Section D Thinking about how you have felt in the past TWO WEEKS, please indicate how strongly you agree or disagree with the following statements:
D1	I have avoided sexual activity to minimise risk of developing or worsening UTI symptoms
D2	I have felt unable to enjoy sexual activity due to my UTI(s)
D3	I have been concerned about the impact of my UTI(s) on my sex life and/or sexual relationship(s)
D4	I have felt that my UTI(s) have made my sexual wellbeing worse
Section E: Patient satisfaction	The following questions are about your feelings of satisfaction with your UTI-related medical care Thinking about how you have felt in the past TWO WEEKS, please indicate how strongly you agree or disagree with the following statements about your UTI-related medical care:
E1	I have generally felt content with the medical care I have been receiving
E2	I have felt confident about being able to get the medical care I need
E3	I have felt like my medical concerns are taken seriously
E4	I have had confidence in the decisions made about my care and treatment
E5	I have felt confident about being able to access UTI testing and treatment quickly enough
E6	I have felt listened to by my healthcare provider(s)
E7	I have had easy access to the medical specialists I need
E8	I have felt like my healthcare provider(s) treat me with respect and dignity
E9	I have been as involved as I have wanted to be in the decisions made about my care and treatment
E10	I have trusted my healthcare provider(s)

A typeset version of the RUTIIQ is available in the online supplementary material (Online Resource 10)

All five sections of the RUTIIQ utilise an 11-point agreement scale ranging from 0 (‘strongly disagree’) to 10 (‘strongly agree’)

^a^The *sexual wellbeing* subscale (Section D) initially asks respondents whether they feel their sexual wellbeing has been impacted by their UTI symptoms in the past two weeks (response options: ‘yes’, ‘no’, ‘prefer not to say’). Respondents who indicate ‘no’ or ‘prefer not to say’ for this question may skip this section and proceed to the final section about satisfaction with UTI-related medical care

The final 30-item RUTIIQ consists of five subscales (see Table 3): *personal wellbeing* (4 items), *social impact* (5 items), *work and activity interference* (7 items), *sexual wellbeing* (4 items), and *patient satisfaction* (10 items). All five sections utilise an 11-point Likert scale ranging from 0 (‘strongly disagree’) to 10 (‘strongly agree’). For the first four subscales, greater scores indicate greater impact to QoL. For the *patient satisfaction* subscale, greater scores indicate greater patient satisfaction with UTI-related medical care.

#### Descriptive statistics and psychometric properties

Observed RUTIIQ subscale scores highlighted the breadth in patient experiences, with scores spanning the full possible range for all subscales except for the *sexual wellbeing* subscale, for which all participants reported at least some level of impact (see Table 4 for RUTIIQ descriptive statistics, see Online Resource 11 for concurrent measure descriptive statistics). The average PHQ-9 and GAD-7 scores indicated moderate depression (*M* = 11.5, *SD* = 7.22) [45], and mild to moderate anxiety (*M* = 9.23, *SD* = 6.21) [46], respectively. Participants typically indicated sexual distress considerably beyond the ‘normal’ FSDS-R range of 0–10 (*M* = 30.3, *SD* = 13.7) [49].

**Table 4:** Descriptive and psychometric statistics of the RUTIIQ

Statistic	RUTIIQ subscale				
	Personal wellbeing	Social wellbeing	Work and activity interference	Sexual wellbeing	Patient satisfaction
Descriptive statistics					
* M*	26.2	23.3	35.8	36.2	36.6
* SD*	11.1	15.9	23.1	6.3	28.5
Range	0–40	0–50	0–70	5–40	0–100
Cronbach’s *α*	.87	.91	.95	.81	.96
ICC	.82	.89	.83	.66	.91
95% CI: LB	.75	.84	.75	.51	.87
95% CI: UB	.87	.92	.88	.78	.94
Spearman’s *ρ*					
PHQ-9	.70**	.69**	.61**	.25**	.26**
GAD-7	.60**	.57**	.46**	.18*	.26**
UCLA-LS	.34**	.53**	.33**	.02	.16*
WPAI:SHP (work domain)	.49**	.61**	.73**	.26**	.28**
WPAI:SHP (activity domain)	.51**	.57**	.70**	.27**	.23**
FSDS-R	.43**	.43**	.34**	.38**	.28**
PSQ-18	− .25**	− .28**	− .35**	− .04	− .76**

*N* = 240 for computation of descriptive statistics, Cronbach’s alpha, and Spearman’s *ρ* for all subscales except for the *sexual wellbeing* subscale due to non-compulsory questions (*N* = 183). *N* = 106 for computation of ICC for all subscales except for the *sexual wellbeing* (*N* = 68)

Maximum possible ranges for RUTIIQ subscales: *personal wellbeing* = 0–40, *social wellbeing* = 0–50, *work and activity interference* = 0–70, *sexual wellbeing* = 0–40, and *patient satisfaction* = 0–100

Cronbach’s alpha (*α*) was selected as a measure of internal consistency. Interpretation: *α* = .80–.90 indicates good internal consistency; *α* = .90–1.00 indicates excellent internal consistency [49]. PROM development guidance recommends minimum Cronbach’s *α* = .70 [22]

ICC (single measures), a measure of test–retest reliability of each subscale between the Baseline and Test–Retest Assessments, was computed for each subscale with a two-way mixed effects model with absolute agreement. Interpretation: ICC = .50–.75 indicates moderate reliability; ICC = .75–.90 indicates good reliability; ICC = .90–1.00 indicates excellent reliability [47]. All ICC were statistically significant at *p* < .001. PROM development guidance recommends minimum ICC = .70 [22]

Spearman’s *ρ* was computed as a measure of construct validity. RUTIIQ *patient satisfaction* subscale scores were reverse scored. The strongest correlation coefficient for each RUTIIQ subscale is in bold. Interpretation of Spearman’s *ρ*: .00 < .10 = negligible, .10–.39 = weak, .40–.69 = moderate, .70–.89 = strong, .90–1.0 = very strong [50]. Statistical significance for construct validity analysis: *p < .05, **p < .01. PROM development guidance recommends minimum Spearman’s *ρ* = .50 [22]

*M* mean, *SD* standard deviation, *ICC* intraclass correlation coefficient, *CI* confidence interval, *LB* lower bound, *UB* upper bound, *RUTIIQ* Recurrent Urinary Tract Infection Impact Questionnaire, *PH*Q-9 Patient Health Questionnaire 9 [45], *GAD-7* Generalized Anxiety Disorder 7 [46], *UCLA-LS* University of Los Angeles Loneliness Scale Version 3 [47], *WPAI:SHP* Work Productivity and Activity Impairment Questionnaire for Specific Health Problems [48], *FSDS-R* Female Sexual Distress Scale–Revised [49], *PSQ-18* Patient Satisfaction Questionnaire 18 [50]

The Automated Readability Index for the RUTIIQ is 6.2, indicating suitability for people with a reading age of 11 years old or above [58]. Internal consistency (Cronbach’s *α*), test–retest reliability (ICC), and construct validity (Spearman’s *ρ*) were moderate to strong for all RUTIIQ subscales (see Table 4). All subscale psychometric statistics surpassed the relevant gold-standard recommendations except the *sexual wellbeing* subscale, which still achieved moderate performance [27, 28].

Linear regression analyses indicated the broad sociocultural applicability of the RUTIIQ, indicating no statistically significant group differences in scores in terms of ethnicity, country of residence, relationship status, or level of fluency in English (*p* > 0.05). Younger respondents typically reported greater rUTI impact than older respondents across all subscales except *sexual wellbeing* (*p* < 0.01, see Online Resource 12). Female participants felt that they experienced greater rUTI impact in *personal wellbeing*, *work and activity interference*, and *sexual wellbeing* than male participants (*p* < 0.05, see Online Resource 13). However, further testing with males is required to examine this difference.

## Discussion

This study developed and validated the first patient-reported outcome measure (PROM) of the psychosocial impact of living with rUTI, the Recurrent UTI Impact Questionnaire (RUTIIQ). The 30-item RUTIIQ provides clinicians and researchers with a unique, critically informed, and patient-evaluated measure of rUTI impact using five key subscales: *personal wellbeing*, *social wellbeing*, *work and activity interference*, *sexual wellbeing*, and *patient satisfaction*. Pilot testing indicated a five-factor structure capable of distinguishing between these concepts, and excellent reliability and validity meeting or exceeding PROM development guidelines [26–28].

The unique strengths of the RUTIIQ development and validation included in-depth input from large, heterogeneous, international samples of patients (*N* = 1983 concept elicitation, *N* = 28 cognitive interviews, *N* = 240 pilot) and expert clinicians (*N* = 15), robustly following gold-standard recommendations and allowing for iterative refinement throughout [26–28]. The demographic and clinical diversity of the patient samples suggests the generalisability of the results across a broad spectrum of rUTI patient experiences. The observed psychometric properties and readability statistics of the RUTIIQ indicate its potential for highly effective application to both clinical and research settings.

Though the research was robustly conducted in accordance with gold-standard PROM development guidelines, some limitations are acknowledged. This research sampled participants from 24 countries, however it is acknowledged that most patient participants were Caucasian, native English-speaking females residing in high-income countries. Additional cross-cultural validation is therefore necessary. Whilst male participants were included, rUTI is more prevalent amongst females [12]; further evaluation of the psychometric properties of the RUTIIQ with males is required. Further test–retest assessment could be conducted with wider time spacing between original completion and retest, following Streiner, Norman & Cairney [43]. Whilst extensive patient involvement was conducted for the development of the RUTIIQ, further qualitative exploration of this measure from the perspective of expert clinicians would be beneficial to facilitate its use in practice. Furthermore, future research could aim to engage caregivers and family members to develop a caregiver-reported adaptation of the RUTIIQ for use in care contexts. The RUTIIQ could also be further validated against other chronic illness measures and populations. Research assessing the clinical responsiveness of the RUTIIQ is ongoing.

The RUTIIQ is the first PROM to specifically assess the patient experience of rUTI psychosocial impact and prioritise the importance of self-report in this unique health context. The UTI Symptom Assessment [61], a symptom-focussed questionnaire for acute UTI, asks respondents to rate how ‘bothersome’ symptoms are, and the Acute Cystitis Symptom Score [62], a symptom-focussed questionnaire for acute cystitis, includes three brief QoL-related questions. The RUTIIQ goes beyond this to capture a fuller picture of the psychosocial challenges faced by this patient cohort, sensitively exploring distinct areas of impact. Unlike generic QoL measures, the RUTIIQ has been developed and validated specifically for use in the rUTI patient population, allowing for evaluation of the unique interactions between psychological, social, and sexual wellbeing reported by patients.

The lack of capacity to measure the rUTI patient experience has been widely reported [23, 25], and the RUTIIQ represents a crucial step towards supplementing well-established clinical testing methods with the patient perspective. Reliable, validated measures of rUTI are essential to improve our understanding of the breadth of challenges associated with this prevalent condition, ultimately improving patient outcomes. Used in conjunction with UTI testing and assessment of symptoms [63], the application of the RUTIIQ in clinical practice allows for standardised observation, patient monitoring, and mapping of patient outcomes [24]. The RUTIIQ enables clinicians to identify how rUTI is impacting their patients, highlighting areas of concern that may require additional support (such as low mood, anxiety, social challenges). This measure has the potential to obtain a sensitive and rapid indication of changes in psychosocial experience over time, and could be used to demonstrate effectiveness of interventions [24].

## Conclusion

The RUTIIQ is an important new outcome measure which specifically evaluates the patient-reported experience of rUTI impact, offering a critical, patient-centred tool for the quantification of psychosocial challenges experienced by this patient cohort. The RUTIIQ has demonstrated strong internal consistency, test–retest reliability, and stability, and has been carefully validated against concurrent measures, producing high construct validity. Such rigorous psychometric validation generates confidence in the use of the RUTIIQ in urology, primary care, and wider healthcare settings. By supporting standardised patient observation and monitoring, clinicians and healthcare professionals are now able to quantitatively calibrate those psychosocial challenges requiring intervention beyond UTI symptom experiences, assessing the effectiveness of their interventions. The RUTIIQ critically prioritises the examination of quality-of-life impact and its uptake will influence both healthcare policy and practice from a patient-centred perspective.

## Supplementary Information

Below is the link to the electronic supplementary material.

Online Resource 1: Framework analysis methodology (Stage I) (PDF 15 kb)

Online Resource 2: Concept elicitation literature review (Stage I) (XLSX 28 kb)

Online Resource 3: Framework analysis results (Stage I)  (DOCX 21 kb)

Online Resource 4: Expert clinician screening results (Stage II) (DOCX 48 kb)

Online Resource 5: Cognitive interview results (Stage III) (DOCX 56 kb)

Online Resource 6: Cognitive interview sampling and recruitment strategy (Stage III) (PDF 69 kb)

Online Resource 7: Cognitive interview topic guide (Stage III) (PDF 59 kb)

Online Resource 8: Pilot sampling and recruitment strategy (Stage IV) (PDF 94 kb)

Online Resource 9: Pilot data handling strategy (Stage IV) (PDF 39 kb)

Online Resource 10: Recurrent Urinary Tract Infection Impact Questionnaire (RUTIIQ) (PDF 157 kb)

Online Resource 11: Pilot descriptive statistics (Stage IV) (DOCX 15 kb)

Online Resource 12: Pilot analysis - age (Stage IV) (DOCX 15 kb)

Online Resource 13: Pilot analysis - gender (Stage IV) (DOCX 15 kb)

## Data Availability

Raw data from this research study and the final Recurrent UTI Impact Questionnaire is archived and accessible in the Open Science Framework data repository: https://osf.io/q2svk (10.17605/OSF.IO/Q2SVK).
