# Supplementary material for: Development and psychometric validation of a patient-reported outcome measure of recurrent urinary tract infection impact: the Recurrent UTI Impact Questionnaire
Source: Qual Life Res. 2023 Feb 6;32(6):1745–58. doi: 10.1007/s11136-023-03348-7 (PMC10172217; doi:10.1007/s11136-023-03348-7)
Supplement: Supplementary file 1 — Online Resource 1: Framework analysis methodology (Stage I) (PDF 15 kb) [file 11136_2023_3348_MOESM1_ESM.pdf]

1. Data familiarisation

- The first author immersed themselves in the data, reviewing all qualitative responses in conjunction with results from a thorough literature review.
- An initial list of key psychosocial factors and recurrent themes was suggested and explored and revised with the whole research team.

2. Identifying a thematic framework

- A thematic framework was devised, including psychosocial 'components' and themes emergent in the data along with known factors from literature.
- 'Subcomponents' were identified for each main 'component' (e.g. 'avoidance' as a subcomponent of 'sexual impact').

3. Indexing

- The thematic framework was systematically applied to the full dataset, using the subcomponents as codes or indexes with which to categorise each qualitative response.
- The first author coded the data, with second coding and triangulation undertaken by the final author.

4. Charting

- To build a picture of the data as a whole, verbatim quotations were arranged according to the appropriate thematic reference and charted alongside other quotations with the same or conceptually similiar codes/indexes.

5. Mapping and interpretation

- The chart of psychosocial factors and supporting quotations was examined to map and interpret the dataset as a whole.
- Associations between subcomponents were assessed and the final concepts were defined and named.
