# Supplementary material for: Development and psychometric validation of a patient-reported outcome measure of recurrent urinary tract infection impact: the Recurrent UTI Impact Questionnaire
Source: Qual Life Res. 2023 Feb 6;32(6):1745–58. doi: 10.1007/s11136-023-03348-7 (PMC10172217; doi:10.1007/s11136-023-03348-7)
Supplement: Supplementary file 3 — Online Resource 3: Framework analysis results (Stage I) (DOCX 21 kb) [file 11136_2023_3348_MOESM3_ESM.docx]

**Online Resource 3.** Thematic framework of the psychosocial impact of rUTI with supporting verbatim quotations

| Framework component | Subcomponent | Verbatim quotations |
| --- | --- | --- |
| Personal wellbeing |  |  |
|  | Depression and low mood | “It massively impacts my mental health. I get extremely depressed and stressed” (UK, 40-49 years old)  “Suicidal thoughts due to UTI, depression that it won’t go away/be cured” (UK, 30-39 years old)  “When I have one it has a dramatic impact on my mood, I feel really low and it impacts on everything” (UK, 40-49 years old) |
|  | Anxiety | “Fear and anxiety about when another occurrence will happen and whether measures to prevent it are good enough” (UK, 30-39 years old)  “The stress of not knowing why they keep occurring, the impact that constant exposure to antibiotics is having on my gut health, and the worry that the infection may become antibiotic resistant” (Australia, 40-49 years old)  “The constant terror over “am I leaking?” “Did I wait five minutes too long to pee because I am not off my floor shift?”” (USA, 30-39 years old) |
|  | Hopelessness | “How hopeless it feels with no solution available. Bladder scans normal and reason cannot be found. This has a huge impact on mental health” (UK, 30-39 years old)  “Fear that there is no cure and that these will permanently incapacitate me and make me unable to work” (USA, 50-59 years old) |
|  | Sleep disruption | “Lack of sleep, I get much worse in the night, depression and a feeling of utter sadness” (UK, 70-79 years old)  “Disrupted sleep impacts my mood during the day” (USA, 60-69 years old) |
| Social wellbeing |  |  |
|  | Isolation and loneliness | “Feeling alone and hopeless” (Germany, 18-24 years old)  “Feeling of isolation as it is not something one can speak about very much” (UK, 40-49 years old)  “Feeling different” (Australia, 18-24 years old) |
|  | Embarrassment and social stigma | “[Impacts on] family as I am embarrassed about it, feel dirty” (USA, 40-49 years old)  “There is a lot of stigma and misunderstanding, people who think you must have unprotected sex or poor hygiene. It’s difficult to explain that during a flare up symptoms can come and go over the course of a week or longer” (UK, 25-29 years old)  “Embarrassment … worried about people’s thoughts due to un-education” (Australia, 18-24 years old) |
|  | Inability to plan and socialise | “I can't leave the house if it’s a bad one. It constantly gets in the way of me making plans and being able to do things as I don’t know when it will happen” (UK, 18-24 years old)  “I’m only 18, I just want to be able to live life without constantly worrying about being on the verge of pain. I want to get dressed up and go out and drink & have fun” (UK, 18-24 years old)  “I think it is such a vicious cycle and can really disrupt your life to the extent that you don't want to plan anything "in case" you get a UTI. It has a really debilitating impact on your life” (Canada, 60-69 years old) |
|  | Social anxiety | “Feel afraid to leave my house because I always feel like I need the loo” (UK, 25-29 years old)  “The difficulties of going out, not knowing where to find toilets” (UK, 60-69 years old) |
| Work and activity interference |  |  |
|  | Poor concentration | “Constant left side and flank pain that is so nagging that I can’t concentrate or function normally” (USA, 60-69 years old)  “Loss of concentration at work because of urge to go to the toilet” (Germany, 30-39 years old) |
|  | Missing work and education | “Ability to go to work. Incontinency and pain means I have to call in sick when I am affected” (UK, 18-24 years old)  “I missed a lot of education during school and university due to UTIs. it is difficult to explain to your boss/colleagues” (UK, 25-29 years old)  “I can’t work” (UK, 25-29 years old) |
|  | Disruption to household and caring responsibilities | “I feel like I can’t leave my house even to do simple things such as a food shop” (UK, 18-24 years old)  “[Impacts on] household chores” (South Africa, 30-39 years old)  “Impact on studies, obligations such as grocery shopping” (Spain, 18-24 years old)  “I'm unable to care for my children while experiencing this pain” (USA, 18-24 years old) |
|  | Financial implications | “Costly due to missing work” (USA, 30-39 years old)  “It really causes issues with my mental health as the financial issues that come alongside are extreme. I'm often too unwell to work and also cannot afford the continuous treatment” (UK, 18-24 years old)  “I pay about 30-40 dollars each time my UTIs become severe enough to require a trip to the doctor. Sometimes I opt out of getting pain meds and only buy antibiotics for sake of pricing and I often delay my trips to the doctors to see if the pain will subside instead of treating the source” (USA, 18-24 years old) |
| Sexual wellbeing |  |  |
|  | Avoidance | “It has made me fearful to have intercourse with my husband. We sometimes wait weeks and then when we do it for 2 days in a row, it seems to come back” (USA, 30-39 years old)  “Have not had sexual activity past 3 months due to UTIs” (USA, 70-79 years old)  “When they only occur when after sex, it's made me seriously consider giving it up. Which means a lonely partnerless life” (USA, 60-69 years old) |
|  | Pain and discomfort | “Pain during sexual activity seems to be quite frequent” (Ireland, 25-29 years old)  “Pain in vagina opening after sex” (UK, 30-39 years old)  “I want to … be able to have sex without pain” (UK, 18-24 years old) |
|  | Anxiety and lack of enjoyment | “I have got to the point where I don't want to have sex, sometimes actively avoid it and enjoy it less because a bladder infection is in the back of my mind” (UK, 25-29 years old)  “The mental health aspect. I tie myself up in knots after having sex because I'm worrying that I'm about to get another UTI, even if I've done everything I can to prevent one” (USA, 40-49 years old)  “Having recurrent UTIs is extremely frustrating, which I think contributes to my mental health and relationship with sex since I get UTIs from having sex” (USA, 25-29 years old) |
|  | Impact on sexual relationships | “Explaining to partner, who thinks I’m avoiding sex because I don’t love him” (UK, 70-79 years old)  “Really want to emphasize the relational and sexual impact. My partner is afraid of having sex with me for fear of my frequent UTIs” (USA, 25-29 years old)  “I want SOLUTIONS to never having one again. So I can be intimate with my husband and express my love and appreciation for him, without living in fear that I’m going to get yet again another UTI from sex” (Canada, 18-24 years old) |
| Patient satisfaction |  |  |
|  | Feeling listened to and taken seriously | “That GP's don’t take the Pain seriously. I have a fairly high pain tolerance, however, this just about brings me undone” (Australia, 50-59 years old)  “For people to have a real understanding of how debilitating they can be/how hard it is to deal with it regularly, and for employers/general public/doctors to take them seriously as a health concern” (UK, 25-29 years old)  “The frustration of doctors who don't listen or believe something is wrong when you describe to them your symptoms” (USA, 18-24 years old)  “I feel misheard and neglected by all doctors I have seen about this. Each UTI is incredibly difficult emotionally because of the stress I have around seeing doctors for this issue and being ignored” (USA, 18-24 years old) |
|  | Feeling dismissed | “The feeling of dismissal by doctors is extremely distressing and under-addressed generally everywhere” (USA, 50-59 years old)  “The fact that doctors don’t really care. They just tell you to pee after sex, and if that doesn’t work they just make it seem like “yep, that’s how it is.”” (Canada, 18-24 years old)  “One of the most frustrating things is being minimized, disregarded or laughed off by doctors that I waste my time and money going to in hopes of help. It is SO frustrating to know my own body; know my own symptoms, do so much research, and still be told that I don't really know what I'm talking about because the test is negative. It is distressing how insensitive some doctors can be to the pain, and how easily they brush off the symptoms as "not real" if the quick pee test doesn't agree” (USA, 50-59 years old) |
|  | Accessibility challenges | “Getting in to see my doctor can be very difficult and sometimes impossible. This is very stressful since I won't get relief without medication. Sometimes have to go to ER” (USA, 70-79 years old)  “Accessibility to antibiotics is a huge one to me, and the reliance on antibiotics for treatment. Suffering from a UTI on a weekend is expensive and dangerous because they escalate so quickly and you don’t have access to tests and antibiotics without a doctor” (Australia, 18-24 years old)  “GPs inability to look at whole picture due to lack of time. This results in being offered short courses of antibiotics which only work for a while” (UK, 60-69 years old) |
|  | Limited clinician understanding of UTI | “I do not think doctors, male or female, understand how seriously ill a UTI can make one feel. They knock me flat for several days or longer. My life is totally disrupted, I have had 10-12 infections in the last 5 1/2 months. That is real and it amounts to an infection almost every other week. The lack of empathy by the medical community is pathetic, and I think there is still a stigma around people saying out loud "I have a bladder infection"” (USA, 60-69 years old)  “Doctors keep telling me it is normal to have a UTI ever 3-6 months because I am a woman, but it doesn't seem normal” (USA, 30-39 years old)  “Not enough GP’s are quick enough to consider the female anatomy and how it could impact on recurrent UTI’s, they are too quick to pass it off as lack of hygiene or practicing safe sex” (UK, 25-29 years old) |

*Note. N* = 1983 female participants. This thematic framework of psychosocial concepts was developed through framework analysis of qualitative survey responses about the rUTI patient experience [23, 24].
