# Supplementary material for: Development and psychometric validation of a patient-reported outcome measure of recurrent urinary tract infection impact: the Recurrent UTI Impact Questionnaire
Source: Qual Life Res. 2023 Feb 6;32(6):1745–58. doi: 10.1007/s11136-023-03348-7 (PMC10172217; doi:10.1007/s11136-023-03348-7)
Supplement: Supplementary file 4 — Online Resource 4: Expert clinician screening results (Stage II) (DOCX 48 kb) [file 11136_2023_3348_MOESM4_ESM.docx]

### **Online Resource 4.** Expert screening study results: content validity indices for items, qualitative feedback, and refinements made

| Original instruction/item | Updated instruction/item | Quotation(s) | Round 1 | | | | Round 2 | | | |
| --- | --- | --- | --- | --- | --- | --- | --- | --- | --- | --- |
|  |  |  | Relevance | | Clarity | | Relevance | | Clarity | |
|  |  |  | *Mdn* | I–CVI | *Mdn* | I–CVI | *Mdn* | I–CVI | *Mdn* | I–CVI |
| Section A |  |  |  |  |  |  |  |  |  |  |
| The following questions relate to the social impact of your UTI(s). | The following questions are about the social impact of your UTI(s). | [grammar/syntax is clearer] | – | – | 6 | 1.00 | – | – | 6 | 1.00 |
| Thinking about how you have felt in the past TWO WEEKS, please indicate how strongly you agree or disagree with the following statements: | – | – | 6 | .93 | 6 | 1.00 | 6 | .92 | 6 | 1.00 |
| Because of my UTI(s)... | – | – | 6 | 1.00 | 6 | 1.00 | 6 | 1.00 | 6 | 1.00 |
| A1. I feel that my ability to form and maintain close relationships with others has been impaired. | – | – | 6 | .93 | 6 | 1.00 | 6 | .92 | 6 | 1.00 |
| A2. I feel that my social activities with other people have been impaired. | – | – | 6 | .93 | 6 | 1.00 | 6 | .92 | 6 | .92 |
| A3. I often feel alone or isolated from others. | – | – | 6 | .93 | 6 | .93 | 6 | .92 | 6 | .92 |
| A4. I often feel left out. | – | – | 6 | 1.00 | 6 | .93 | 6 | 1.00 | 6 | .92 |
| A5. I feel that I am no longer close to anyone. | – | – | 6 | 1.00 | 6 | .93 | 6 | 1.00 | 6 | .92 |
| A6. I often feel different from others. | – | – | 6 | .93 | 6 | 1.00 | 6 | 1.00 | 6 | 1.00 |
| A7. I often feel worried that I am a burden to others. | – | – | 6 | .93 | 6 | 1.00 | 6 | 1.00 | 6 | 1.00 |
| A8. I often feel embarrassed in social situations. | – | – | – | – | 6 | 1.00 | – | – | 6 | 1.00 |
| A9. I often feel anxious in social situations. | – | – | 6 | .93 | 6 | 1.00 | 6 | .92 | 6 | 1.00 |
| Scale: 0 = strongly disagree; 10 = strongly agree | – | – | – | – | – | – | – | – | – | – |
| NEW ITEM | I avoid socialising more than I used to. | GP (Canada): “activity avoidance is an issue”  Urogynaecologist (USA): “I felt like a lot of the questions asked were doing more of a job of assessing depressive type symptoms, not necessarily the range of fear and guilt and withdrawal and self–blame that can be so devastating.” | – | – | – | – | – | – | – | – |

*Note.* *Mdn* = Median rating. I–CVI = item content validity index.

Round 1 *n* = 15. Round 2 *n* = 12.

Instructions were tested only for clarity of wording. Items were tested for both clarity and relevance for recurrent UTI.

Median ratings < 4 are in bold. I–CVI < .75 are in bold. I–CVI = 1.00 indicates that all expert participants rated the item/instruction as at least 4 out of 6 (where 6 = highly relevant/clear).

Updated items were taken forward for testing in the first phase of patient cognitive interviews. Hyphens (−) indicate that no relevance or clarity ratings were obtained (for example, for new items or instructions added after expert screening, or for instructions which were only tested for clarity), or that no changes were made.

| Original instruction/item | Updated instruction/item | Quotation(s) | Round 1 | | | | Round 2 | | | |
| --- | --- | --- | --- | --- | --- | --- | --- | --- | --- | --- |
|  |  |  | Relevance | | Clarity | | Relevance | | Clarity | |
|  |  |  | *Mdn* | I–CVI | *Mdn* | I–CVI | *Mdn* | I–CVI | *Mdn* | I–CVI |
| Section B |  |  |  |  |  |  |  |  |  |  |
| The following questions relate to the impact of your UTI(s) on your work. The term "work" is used here to include paid or unpaid employment, and/or regular daily activities, such as home management or caring responsibilities, studying, etc. | – | – | – | – | 6 | 1.00 | – | – | 6 | 1.00 |
| Thinking about how you have felt in the past TWO WEEKS, due to your UTI(s), please indicate how often: |  |  |  |  |  |  |  |  |  |  |
| B1. Your ability to work was impaired. | – | – | 6 | 1.00 | 6 | .93 | 6 | 1.00 | 6 | .92 |
| B2. You missed full or partial days of work, studying or home responsibilities | – | – | 6 | 1.00 | 6 | 1.00 | 6 | 1.00 | 6 | 1.00 |
| B3. The kind or amount of work you could do was limited. | – | – | 6 | 1.00 | 6 | 1.00 | 6 | 1.00 | 6 | 1.00 |
| B4. It was difficult to concentrate on your work. | It was more difficult than usual to concentrate on your work. | [as below for B5] | 6 | .93 | 6 | 1.00 | 6 | .89 | 6 | 1.00 |
| B5. It was difficult to handle your workload. | It was more difficult than usual to handle your workload. | GP (UK): “more difficult than usual to handle your workload?” | 6 | .93 | 6 | .93 | 6 | .91 | 6 | 1.00 |
| B6. The quality of your work was lower than it should have been. | The quality of your work was lower than usual. | Urogynaecologist (USA): “"it should have been" is weighted with many ideas. Maybe something like "that you wanted it to be."” | 6 | .93 | 6 | .93 | 6 | .92 | 6 | .92 |
| B7. You experienced financial pressure. | You experienced financial pressure (e.g. due to medical costs, missing work). | Urogynaecologist (USA): “What is "financial pressure?" Means very different things to different people.”  Urogynaecologist (USA): “unclear – non–specific” | 6 | **.73** | 6 | .80 | 6 | .83 | 6 | .92 |
| Scale: 0 = never; 10 = always | – | – | – | – | – | – | – | – | – | – |

*Note.* *Mdn* = Median rating. I–CVI = item content validity index.

Round 1 *n* = 15. Round 2 *n* = 12.

Instructions were tested only for clarity of wording. Items were tested for both clarity and relevance for recurrent UTI.

Median ratings < 4 are in bold. I–CVI < .75 are in bold. I–CVI = 1.00 indicates that all expert participants rated the item/instruction as at least 4 out of 6 (where 6 = highly relevant/clear).

Updated items were taken forward for testing in the first phase of patient cognitive interviews. Hyphens (−) indicate that no relevance or clarity ratings were obtained (for example, for new items or instructions added after expert screening, or for instructions which were only tested for clarity), or that no changes were made.

| Original instruction/item | Updated instruction/item | Quotation(s) | Round 1 | | | | Round 2 | | | |
| --- | --- | --- | --- | --- | --- | --- | --- | --- | --- | --- |
|  |  |  | Relevance | | Clarity | | Relevance | | Clarity | |
|  |  |  | *Mdn* | I–CVI | *Mdn* | I–CVI | *Mdn* | I–CVI | *Mdn* | I–CVI |
| Section C |  |  |  |  |  |  |  |  |  |  |
| The following questions are about pain during or after sexual activity due to your UTI(s). | The following questions are about pain during or after sexual activity due to your UTI(s). Please consider both penetrative and non–penetrative sexual activity involving the genital and/or anal areas. | GP (UK): “Sexually active covers a myriad of activities, these patients will typically be triggered into a flare up via penetrative sexual intercourse but may be able to carry out non penetrative activities without consequence”  Urogynaecologist (USA): “May want to define sexual activity (any? Or only penetrative Intercourse?)”  GP (USA): “Clarify is sex just penile–vaginal intercourse or does it include oral sex, non–heterosexual sex?” | – | – | 6 | 1.00 | – | – | 6 | 1.00 |
| Have you been sexually active in the past two weeks? | Have you engaged in sexual activity in the past two weeks? | GP (USA): “Any sexual activity? If can clarify, that would be good.”  GP (USA): “(If sexual activity has been previously defined)”  Urologist (USA): “Prefer "Have you engaged in sexual activity" – some people feel that "have you been sexually active" is sort of like being promiscuous – so I have learned to modify how I ask this. It's a generational thing.” | – | – | 6 | **.71** | – | – | 6 | .75 |
| Responses: Yes; No |  |  |  |  |  |  |  |  |  |  |
| If you selected “No”, please skip to section D. If you selected “Yes”, please continue with this section. |  |  |  |  |  |  |  |  |  |  |
| Thinking about how you have felt in the past TWO WEEKS, please indicate how often you experienced any pain: | Thinking about how you have felt in the past TWO WEEKS, please indicate how OFTEN you experienced any lower abdominal, genital or bladder pain: | GP (USA): “pain where?”  GP (UK): “Think this should be justified by relating the pain to urinary tract not just 'pain'” |  |  |  |  |  |  |  |  |
| C1. During sexual activity | – | – | 6 | .93 | 6 | .93 | 6 | 1.00 | 6 | .92 |
| C2. After sexual activity | Within the 48 hours after sexual activity | GP (UK): “The only improvement I could suggest is popping a couple of time frames in e.g. the next day / after a couple of days” | 6 | .93 | 6 | .93 | 6 | 1.00 | 6 | 1.00 |
| Scale: 0 = never; 10 = always | – | – | – | – | – | – | – | – | – | – |
| Thinking about how you have felt in the past TWO WEEKS, please indicate your average level of pain: | Thinking about how you have felt in the past TWO WEEKS, please indicate whether you experienced any lower abdominal, genital or bladder pain and how SEVERE it was: | GP (USA): “pain where?”  GP (UK): “Think this should be justified by relating the pain to urinary tract not just 'pain'”  [wording in line with RUTISS] | – | – | 6 | .87 | – | – | 6 | .92 |
| C3. During sexual activity | – | – | 6 | .87 | 6 | 1.00 | 6 | .92 | 6 | 1.00 |
| C4. After sexual activity | Within the 48 hours after sexual activity | [as above for C2] | 6 | .93 | 6 | 1.00 | 6 | 1.00 | 6 | 1.00 |
| Scale: 0 = no pain; 1 = very mild; 10 = worst imaginable pain | – | – | – | – | – | – | – | – | – | – |

*Note.* *Mdn* = Median rating. I–CVI = item content validity index.

Round 1 *n* = 15. Round 2 *n* = 12.

Instructions were tested only for clarity of wording. Items were tested for both clarity and relevance for recurrent UTI.

Median ratings < 4 are in bold. I–CVI < .75 are in bold. I–CVI = 1.00 indicates that all expert participants rated the item/instruction as at least 4 out of 6 (where 6 = highly relevant/clear).

Updated items were taken forward for testing in the first phase of patient cognitive interviews. Hyphens (−) indicate that no relevance or clarity ratings were obtained (for example, for new items or instructions added after expert screening, or for instructions which were only tested for clarity), or that no changes were made.

| Original instruction/item | Updated instruction/item | Quotation(s) | Round 1 | | | | Round 2 | | | |
| --- | --- | --- | --- | --- | --- | --- | --- | --- | --- | --- |
|  |  |  | Relevance | | Clarity | | Relevance | | Clarity | |
|  |  |  | *Mdn* | I–CVI | *Mdn* | I–CVI | *Mdn* | I–CVI | *Mdn* | I–CVI |
| Section D |  |  |  |  |  |  |  |  |  |  |
| The following questions relate to the sexual impact of your UTI(s). | – | – | – | – | 6 | 1.00 | – | – | 6 | .92 |
| Do you feel your UTI(s) has/have impacted you sexually in the past two weeks? | Do you feel your UTI(s) has/have impacted your sex life in the past two weeks? | GP (UK): “impacted on your sex life' rather than 'impacted you sexually'?” |  |  |  |  |  |  |  |  |
| Responses: Yes; No | – | – | – | – | – | – | – | – | – | – |
| If you selected “No”, please skip to section E. If you selected “Yes”, please continue with this section. |  |  |  |  |  |  |  |  |  |  |
| Thinking about how you have felt in the past TWO WEEKS, please indicate how strongly you agree or disagree with the following statements: | – | – | – | – | 6 | 1.00 | – | – | 6 | 1.00 |
| Thinking of my UTI(s)… | [remove and integrate within items] | Urogynaecologist (USA): “The statement "thinking of my UTIs, I feel able to enjoy myself sexually." is a bit strange. Important domain to get at, but need to focus the question better to say something more like "I am able to enjoy myself sexually without fear of UTI" or something."  Urologist (USA): “The stem "thinking about my UTI(s) doesn't read correctly.” |  |  |  |  |  |  |  |  |
| D1. I feel content with the way my sex life is presently. | REMOVE ITEM | Urologist (USA): “This is a vague question. better to ask if sex avoidance due to fear of UTI” | 6 | .93 | 6 | .93 | 6 | 1.00 | 6 | 1.00 |
| D2. I feel able to enjoy myself sexually. | I feel unable to enjoy sexual activity due to my UTI(s). | [as per integration after removing “thinking of my UTI(s)…”]  Urogynaecologist (USA): “Always weird to use questions that go in different directions on the agree/disagree scale for a single condition. Sometimes people get confused and answer wrong.” | 6 | .93 | 6 | .87 | 6 | 1.00 | 6 | 1.00 |
| D3. I am concerned about its impact on my sex life. | I am concerned about the impact of my UTI(s) on my sex life. | [as per integration after removing “thinking of my UTI(s)…”] | 6 | 1.00 | 6 | .93 | 6 | 1.00 | 6 | 1.00 |
| D4. I feel that my sexual wellbeing is affected. | I feel that my UTI(s) have made my sexual wellbeing worse. | [as per integration after removing “thinking of my UTI(s)…”]  [grammar/vague – no direction in “affected”] | 6 | 1.00 | 6 | 1.00 | 6 | 1.00 | 6 | 1.00 |
| Scale: 0 = strongly disagree; 10 = strongly agree | – | – | – | – | – | – | – | – | – | – |
| NEW ITEM | [to come first and replace the current D1]  I avoid sexual activity to minimise risk of UTI symptoms. | Urogynaecologist (USA): “The statement "thinking of my UTIs, I feel able to enjoy myself sexually." is a bit strange. Important domain to get at, but need to focus the question better to say something more like "I am able to enjoy myself sexually without fear of UTI" or something." | – | – | – | – | – | – | – | – |

*Note.* *Mdn* = Median rating. I–CVI = item content validity index.

Round 1 *n* = 15. Round 2 *n* = 12.

Instructions were tested only for clarity of wording. Items were tested for both clarity and relevance for recurrent UTI.

Median ratings < 4 are in bold. I–CVI < .75 are in bold. I–CVI = 1.00 indicates that all expert participants rated the item/instruction as at least 4 out of 6 (where 6 = highly relevant/clear).

Updated items were taken forward for testing in the first phase of patient cognitive interviews. Hyphens (−) indicate that no relevance or clarity ratings were obtained (for example, for new items or instructions added after expert screening, or for instructions which were only tested for clarity), or that no changes were made.

| Original instruction/item | Updated instruction/item | Quotation(s) | Round 1 | | | | Round 2 | | | |
| --- | --- | --- | --- | --- | --- | --- | --- | --- | --- | --- |
|  |  |  | Relevance | | Clarity | | Relevance | | Clarity | |
|  |  |  | *Mdn* | I–CVI | *Mdn* | I–CVI | *Mdn* | I–CVI | *Mdn* | I–CVI |
| Section E |  |  |  |  |  |  |  |  |  |  |
| The following questions relate to your feelings of patient satisfaction as someone who experiences UTIs. | The following questions relate to your feelings of satisfaction with your UTI-related medical care. | GP (UK): “Medical care for UTI’s or care in general?”  Urogynaecologist (USA): “Add ‘for my UTIs’” | – | – | 6 | 1.00 | – | – | 6 | .92 |
| Thinking about how you have felt in the past TWO WEEKS, please indicate how strongly you agree or disagree with the following statements: | Thinking about how you have felt in the past TWO WEEKS, please indicate how strongly you agree or disagree with the following statements about your UTI-related medical care: | [as above] |  |  |  |  |  |  |  |  |
| E1. I feel content with the medical care I am receiving. | – | – | 6 | 1.00 | 6 | 1.00 | 6 | 1.00 | 6 | 1.00 |
| E2. I feel confident I can get the medical care I need. | – | – | 6 | 1.00 | 6 | .93 | 6 | 1.00 | 6 | .92 |
| E3. I am treated with respect and dignity by my medical healthcare provider(s). | – | – | 6 | 1.00 | 6 | .93 | 6 | 1.00 | 6 | .92 |
| E4. I feel like my medical concerns are taken seriously. | – | – | 6 | 1.00 | 6 | 1.00 | 6 | 1.00 | 6 | 1.00 |
| E5. I feel listened to by my medical healthcare provider(s). | – | – | 6 | 1.00 | 6 | 1.00 | 6 | 1.00 | 6 | 1.00 |
| E6. I have confidence in the decisions made about my care and treatment. | – | – | 6 | 1.00 | 6 | .93 | 6 | 1.00 | 6 | .92 |
| E7. I have been as involved as I have wanted to be in the decisions made about my care and treatment. | – | – | 6 | .93 | 6 | .93 | 6 | .92 | 6 | .92 |
| E8. I trust my medical healthcare provider(s). | – | – | 6 | 1.00 | 6 | 1.00 | 6 | 1.00 | 6 | 1.00 |
| E9. I have easy access to the medical specialists I need. | – | – | 6 | 0.93 | 6 | .93 | 6 | .92 | 6 | .92 |
| Scale: 0 = strongly disagree; 10 = strongly agree | – | – | – | – | – | – | – | – | – | – |

*Note.* *Mdn* = Median rating. I–CVI = item content validity index.

Round 1 *n* = 15. Round 2 *n* = 12.

Instructions were tested only for clarity of wording. Items were tested for both clarity and relevance for recurrent UTI.

Median ratings < 4 are in bold. I–CVI < .75 are in bold. I–CVI = 1.00 indicates that all expert participants rated the item/instruction as at least 4 out of 6 (where 6 = highly relevant/clear).

Updated items were taken forward for testing in the first phase of patient cognitive interviews. Hyphens (−) indicate that no relevance or clarity ratings were obtained (for example, for new items or instructions added after expert screening, or for instructions which were only tested for clarity), or that no changes were made.
