# Supplementary material for: Development and psychometric validation of a patient-reported outcome measure of recurrent urinary tract infection impact: the Recurrent UTI Impact Questionnaire
Source: Qual Life Res. 2023 Feb 6;32(6):1745–58. doi: 10.1007/s11136-023-03348-7 (PMC10172217; doi:10.1007/s11136-023-03348-7)
Supplement: Supplementary file 5 — Online Resource 5: Cognitive interview results (Stage III) (DOCX 56 kb) [file 11136_2023_3348_MOESM5_ESM.docx]

### **Online Resource 5.** Cognitive interview study results: qualitative feedback and refinements made after the first phase of patient cognitive interviewing

| Original instruction/item | Updated instruction/item | Code(s) | Quotation(s) |
| --- | --- | --- | --- |
| Section A |  |  |  |
| The following questions are about the social impact of your UTI(s). | – | – | – |
| Thinking about how you have felt in the past TWO WEEKS, please indicate how strongly you agree or disagree with the following statements: | – | – | – |
| Because of my UTI(s)… | – | – | – |
| A1. I feel that my ability to form and maintain close relationships with others has been impaired. | Changed to PAST TENSE.  Split into two questions:  1. I have felt that my close relationships have been impaired.  2. I have felt that my ability to form new close relationships has been impaired. | Double-barrelled,  Other | TENSE CHANGE:  24-year-old, female, UK: “I suppose I kind of forgot about the past two weeks thing, and I was just kind going about it in general. But yeah, all of that is still relevant to the past two weeks, because it is just kind of just stuff I deal with all the time”  48-year-old, female, USA: “That one's a bit confusing. Does that mean in the past two weeks? I often feel alone. Not in general … If it said I have felt lonely or isolated, it could be language and grammar there”  SPLIT:  53-year-old, female, UK: “Close relationships, depends. For not, like for friends and family, I'd say no, but for intimate relationships, definitely. I think there's a difference between maintaining close relationships with, with people I've known all my life and potentially meeting new people, I'm single, so if it's impaired, if it's impacted on anything. It's impacted on me not, not seeking out an intimate relationship … Form and maintain, maybe there’s two different questions there. I would say a big fat ten for form, and a one or two [for] maintain”  56-year-old, female, UK: “Well, does that mean, like new people? So if I'm forming a relationship, so presumably, it's getting in the way of me making new relationships” |
| A2. I feel that my social activities with other people have been impaired. | Changed to PAST TENSE: I have felt that my social activities with others have been impaired. | Other | [as above in A1] |
| A3. I often feel alone or isolated from others. | Changed to PAST TENSE: I have felt alone or isolated from others. | Other | [as above in A1] |
| A4. I often feel left out. | Changed to PAST TENSE: I have felt left out. | Other | [as above in A1] |
| A5. I feel that I am no longer close to anyone. | Changed to PAST TENSE: I have felt that I am no longer close to anyone. | Other | [as above in A1] |
| A6. I often feel different from others. | Changed to PAST TENSE: I have felt different from others. | Other | [as above in A1] |
| A7. I often feel worried that I am a burden to others. | Changed to PAST TENSE: I have felt worried that I am a burden to others. | Other | [as above in A1] |
| A8. I often feel embarrassed in social situations. | Order change: avoidance (A10) to come before embarrassment (A8) and anxiety (A9).  Changed to PAST TENSE: I have felt embarrassed in social situations. | Clarity: Vague,  Other | [as above in A1]  ORDER CHANGE:  24-year-old, female, UK: “if I’m that bad, I wouldn’t leave the house”  36-year-old, non-binary, USA: “If I’m having an episode, I don’t put myself in social situations … I would just avoid [them]” |
| A9. I often feel anxious in social situations. | Order change: avoidance (A10) to come before embarrassment (A8) and anxiety (A9).  Changed to PAST TENSE: I have felt anxious in social situations. | Clarity: Vague,  Other | [as above in A8]  [as above in A1] |
| A10. I avoid socialising more than I used to. | Order change: avoidance (A10) to come before embarrassment (A8) and anxiety (A9).  Changed to PAST TENSE: I have avoided socialising more than I used to. | Clarity: Vague,  Other | [as above in A8]  [as above in A1] |
| Scale: 0 = strongly disagree; 10 = strongly agree | – | – | – |

*Note.* Code(s) from the Question Appraisal System – 99 [42]. Items are written as they are presented in the measure (i.e., with underlined or bold phrasing). Updated items were taken forward for testing in the second phase of patient cognitive interviews.

Hyphens (−) indicate that no changes were made to the corresponding item.

| Original instruction/item | Updated instruction/item | Code(s) | Quotation(s) |
| --- | --- | --- | --- |
| Section B |  |  |  |
| The following questions are about the impact of your UTI(s) on your work. The term "work" is used here to include paid or unpaid employment, and/or regular daily activities, such as home management or caring responsibilities, studying, etc. | – | – | – |
| Thinking about how you have felt in the past TWO WEEKS, due to your UTI(s), please indicate how often: | – | – | – |
| B1. Your ability to work was impaired. | Order change: Missing work (B2) to come before impairment (B1)  Changed to first person: My ability to work was impaired. | Clarity: Vague,  Other | [Person change in line with rest of RUTIIQ] |
| B2. You missed full or partial days of work, home responsibilities or studying. | Order change: Missing work (B2) to come before impairment (B1)  Changed to first person: I have regularly missed full or partial days of work, home responsibilities or studying. | Clarity: Vague,  Other | [Person change in line with rest of RUTIIQ] |
| B3. The kind or amount of work you could do was limited. | Changed to first person: The kind or amount of work I could do was limited. | Other | [Person change in line with rest of RUTIIQ] |
| B4. It was more difficult than usual to concentrate on your work. | Changed to first person: It was more difficult than usual to concentrate on my work. | Other | [Person change in line with rest of RUTIIQ] |
| B5. It was more difficult than usual to handle your workload. | Changed to first person: It was more difficult than usual to handle my workload. | Other | [Person change in line with rest of RUTIIQ] |
| B6. The quality of your work was lower than usual. | Changed to first person: The quality of my work was lower than usual. | Other | [Person change in line with rest of RUTIIQ] |
| B7. You experienced financial pressure (e.g. due to medical costs, missing work). | Changed to first person: I experienced financial pressure (e.g. due to medical costs, missing work). | Other | [Person change in line with rest of RUTIIQ] |
| Scale: 0 = never; 10 = always | 0 = strongly disagree; 10 = strongly agree | Responses: Mismatch | 56-year-old, female, UK: “So if I said six times, would that mean six, would that mean six times? … the words How often leads me to think how many times … What’s a partial day? Is that a one?”  32-year-old, female, United Arab Emirates: “I'd missed like two days of work in the last two weeks. Where would I rank myself on a zero to 10 With that? Does 10 mean that I would have had to have missed every day of work from the last two weeks? Or, and or do I consider missing two days is bad enough to warrant a ten?” |
| NEW ITEM | [to come between impairment (B1) and kind/amount of work limited (B3)]  I have regularly put pressure on myself to work despite feeling unwell. | – | 56-year-old, female, UK: “I think the big thing for me was, is that you go into work anyway … putting pressure on yourself to do something that actually you’re really not properly well enough to do … if you felt that ill on something you got as a one–off, you probably wouldn’t go to work. But because you get it all the time, you feel, oh God, I can’t. You know? I can’t not go to work. Or you don’t want any colleagues thinking, again? I’ve got, I’ve got another one”  30-year-old, female, Canada: “If I gave that a low ranking, it makes it seem as if the quality–of–life impact is less than it actually is. Like, just because I am able to deliver … it doesn’t mean that it diminishes my experience”  53-year-old, female, UK: “I'm not really... there have been times where I probably should have because I haven't been well, but I'm just not one for taking time off sick from work. I just try to power on through. So, so I haven't missed any work because of it”  48-year-old, female, USA: “I've just sort of said that I do feel like it's impacted me quite a lot. But then when I say that, say that, I didn't miss any full days” |

*Note.* Code(s) from the Question Appraisal System – 99 [42]. Items are written as they are presented in the measure (i.e., with underlined or bold phrasing). Updated items were taken forward for testing in the second phase of patient cognitive interviews.

Hyphens (−) indicate that no changes were made to the corresponding item.

| Original instruction/item | Updated instruction/item | Code(s) | Quotation(s) |
| --- | --- | --- | --- |
| Section C | Order change: Sexual pain (C) to come after sexual impact (D) | Illogical order | 71-year-old, female, USA: “The question is, has the UTIs, recurrent UTIs affected your sexual, you know, activity? … The answer is yes, it has, but not anymore because there’s no sexual activity anymore … the question should be if you don’t engage in any sexual activity, is it because of fear of UTI?” |
| The following questions are about pain during or after sexual activity due to your UTI(s). Please consider both penetrative and non–penetrative sexual activity involving the genital and/or anal areas. | The following questions are about pain during or after sexual activity due to your UTI(s). Please consider both penetrative and non–penetrative sexual activity involving the genital and/or anal areas, with or without a partner(s). | Clarity: Vague | 24-year-old, female, UK: “I automatically assumed that it would have been with a partner … but it doesn’t say with a partner, it just says sexual activity, you can have sexual activity with yourself … alone or with partners or something like that would make that more clear”  71-year-old, female, USA: “I would think that somebody may not think of, you know, masturbation, they may not think of that” |
| Have you engaged in sexual activity in the past two weeks? | – | – | – |
| Responses: Yes; No | Responses: Yes; No; Prefer not to say | Sensitive content | 18-year-old, female, USA: “Yeah, let’s skip ahead”  57-year-old, male, UK: “The whole, the whole issue, what I'm worried about it is the whole thing about, you know, masturbation and mentioning, mentioning anal sex and genitals, you know, a lot of people are gonna freak out. Especially in this part of the world, there's quite a lot, kind of, you know, DUP people and you know. That's kinda... But no, I mean, it doesn't annoy me in the least, I'm happy to answer them all, But I can see it being a difficult question for some people … a lot of people couldn’t answer it at all” |
| If you selected “No”, please skip to section D. If you selected “Yes”, please continue with the rest of Section C. | If you selected “No” or “Prefer not to say”… | Sensitive content | [See above] |
| Thinking about how you have felt in the past TWO WEEKS, please indicate how OFTEN you experienced any lower abdominal, genital or bladder pain: | Thinking about how you have felt in the past TWO WEEKS, please indicate how strongly you agree or  disagree with the following statements about your lower abdominal, genital and/or bladder pain or  discomfort: | Responses: Mismatch,  Clarity: Vague | [See participant 32-year-old, female, United Arab Emirates quotation for scale update below] |
| C1. During sexual activity. | I have experienced pain or discomfort here during sexual activity. | Other | [to match the updated instruction above] |
| C2. Within the 48 hours after sexual activity. | I have experienced pain or discomfort here within the 48 hours after sexual activity. | Other | [to match the updated instruction above] |
| Scale: 0 = never; 10 = always | 0 = strongly disagree; 10 = strongly agree | Responses: Mismatch | 32-year-old, female, United Arab Emirates: “I don't know where I'd put myself. Because if I had sex three times let's say and then twice, there was no pain, and once there was, where would I put myself on a 10 point scale on that?” |
| Thinking about how you have felt in the past TWO WEEKS, please indicate whether you experienced any lower abdominal, genital or bladder pain and how SEVERE it was: | If you experienced lower abdominal, genital and/or bladder pain or discomfort related to sexual activity in the past TWO WEEKS, please indicate how SEVERE it was: | Conflicting instructions | 36-year-old, female, Canada: “What’s the difference between the [C1/2 and C3/4] questions? … So the top one is saying how often you’ve had these symptoms, and then the bottom, you want to know how severe … essentially we’ve already answered if we’ve experienced it in the first [two], so maybe this one can say … if you have experienced, how severe was it?”  30-year-old, female, Canada: “Wait, sorry. I’m kind of trying to understand the difference between the first [two] and the second [two] ... Oh, so… sorry, I totally misinterpreted the first [two as severity questions] then”  57-year-old, male, UK: “Is that the same question?” |
| C3. During sexual activity. | – | – | – |
| C4. Within the 48 hours after sexual activity. | – | – | – |
| Scale: 0 = not present; 1 = very mild; 10 = worst imaginable pain | 0 = not present; 1 = very mild; 10 = extremely severe | Responses: Mismatch | [to match the updates in RUTISS symptom and pain severity scale response options] |

*Note.* Code(s) from the Question Appraisal System – 99 [42]. Items are written as they are presented in the measure (i.e., with underlined or bold phrasing). Updated items were taken forward for testing in the second phase of patient cognitive interviews.

Hyphens (−) indicate that no changes were made to the corresponding item.

| Original instruction/item | Updated instruction/item | Code(s) | Quotation(s) |
| --- | --- | --- | --- |
| Section D | Order change: Sexual pain (C) to come after sexual impact (D) |  |  |
| The following questions relate to the sexual impact of your UTI(s). | – | – | – |
| Do you feel your UTI(s) has/have impacted your sex life in the past two weeks? | – | – | – |
| Responses: Yes; No | Responses: Yes; No; Prefer not to say | Sensitive content | [as in Section C] |
| If you selected “No”, please skip to section E. If you selected “Yes”, please continue with the rest of Section D. | If you selected “No” or “Prefer not to say”… | Sensitive content | [as in Section C] |
| Thinking about how you have felt in the past TWO WEEKS, please indicate how strongly you agree or disagree with the following statements: | – | – | – |
| D1. I avoid sexual activity to minimise risk of UTI symptoms. | Changed to PAST TENSE: I have avoided sexual activity to minimise risk of developing or worsening of UTI symptoms. | Other | [Tense change in line with the rest of RUTIIQ] |
| D2. I feel unable to enjoy sexual activity due to my UTI(s). | Changed to PAST TENSE: I have felt unable to enjoy sexual activity due to my UTI(s). | Other | [Tense change in line with the rest of RUTIIQ] |
| D3. I am concerned about the impact of my UTI(s) on my sex life. | Changed to PAST TENSE: I have been concerned about the impact of my UTI(s) on my sex life and/or sexual relationships. | Clarity: Vague,  Other | [Tense change in line with the rest of RUTIIQ]  56-year-old, female, UK: “I think it’s the impact on the relationship because of the, because of the impact that it can have on making you feel a little bit reluctant”  36-year-old, female, Canada: “I don’t know if it’s, I’m concerned about my impact, the impact of my UTIs on my sex life, or if it’s more, I’m concerned about it on my relationship with my partner or lover”  26-year-old, female, Austria: “It’s definitely an impact on it, and that spills over into the relationship. I think it’s not really just the sex life thing, but how it spills over, and in my past relationship that I had during these really harsh episodes, we just drifted apart … first the sex stopped and then the emotional connection just kind of went” |
| D4. I feel that my UTI(s) have made my sexual wellbeing worse. | Changed to PAST TENSE: I have felt that my UTI(s) have made my sexual wellbeing worse. | Other | [Tense change in line with the rest of RUTIIQ] |
| Scale: 0 = strongly disagree; 10 = strongly agree | – | – | – |

*Note.* Code(s) from the Question Appraisal System – 99 [42]. Items are written as they are presented in the measure (i.e., with underlined or bold phrasing). Updated items were taken forward for testing in the second phase of patient cognitive interviews.

Hyphens (−) indicate that no changes were made to the corresponding item.

| Original instruction/item | Updated instruction/item | Code(s) | Quotation(s) |
| --- | --- | --- | --- |
| Section E |  |  |  |
| The following questions relate to your feelings of satisfaction with your UTI–related medical care. | – | – | – |
| Thinking about how you have felt in the past TWO WEEKS, please indicate how strongly you agree or disagree with the following statements about your UTI–related medical care: | – | – | – |
| E1. I feel content with the medical care I am receiving. | Changed to PAST TENSE: I have generally felt content with the medical care I have been receiving. | Other | [Tense change in line with the rest of RUTIIQ] |
| E2. I feel confident I can get the medical care I need. | Changed to PAST TENSE: I have felt confident about being able to get the medical care I need. | Other | [Tense change in line with the rest of RUTIIQ] |
| E3. I am treated with respect and dignity by my medical healthcare provider(s). | Order change: to come below seriously (E4) and listened to (E5).  Changed to PAST TENSE: I have felt like my healthcare provider(s) treat me with respect and dignity. | Knowledge,  Other | [Tense change in line with the rest of RUTIIQ]  ORDER CHANGE:  24-year-old, female, UK: “[to E4] It’s never been taken seriously at all … telling me I don’t have an infection when I do, and just basically blaming it on me, saying it’s all to do with my lifestyle choices and all that like, just totally, just not being treated with respect” |
| E4. I feel like my medical concerns are taken seriously. | Changed to PAST TENSE: I have felt like my medical concerns are taken seriously. | Other | [Tense change in line with the rest of RUTIIQ] |
| E5. I feel listened to by my medical healthcare provider(s). | Changed to PAST TENSE: I have felt listened to by medical healthcare provider(s). | Other | [Tense change in line with the rest of RUTIIQ] |
| E6. I have confidence in the decisions made about my care and treatment. | Changed to PAST TENSE: I have had confidence in the decisions made about my care and treatment. | Other | [Tense change in line with the rest of RUTIIQ] |
| E7. I have been as involved as I have wanted to be in the decisions made about my care and treatment. | – | – | – |
| E8. I trust my medical healthcare provider(s). | Changed to PAST TENSE: I have trusted my healthcare provider(s). | Other | [Tense change in line with the rest of RUTIIQ] |
| E9. I have easy access to the medical specialists I need. | Order change: to come before seriously (E4)  Changed to PAST TENSE: I have had easy access to the medical specialists I need. | Other | [Tense change in line with the rest of RUTIIQ] |
| Scale: 0 = strongly disagree; 10 = strongly agree | – | – | – |
| NEW ITEM | [to come after E2]  I have felt confident about being able to access UTI testing and treatment quickly enough. | – | 57-year-old, male, UK: “it can happen quite rapidly, you can being going along nicely one day, nothing, and then I mean, probably, I've had a few instances where I've been away, maybe just maybe drove to [local city] for the day, which is like a two hour drive back and you're fine, there's nothing wrong. You get home, go to the bathroom and go, Ah shit. It's a real sting. And this is a Friday afternoon. Yeah, GPs closed, and you're thinking, Now what do I do? I'm not gonna leave this until the morning, because this is stinging like hell now. And then to try to get an out of hours doctor, he's going to give you a prescription and then there's no process for it. That's, that's why I, when you get through to them on the phone it works fine, it's fine, and the process, I think, works well. But it's the access to it now and the time you do it, and the timescales involved”  75-year-old, female, South Africa: “I don’t know how … how quickly one can get medication, whether one can go down to your local hospital to get an antibiotic … I was caught so many times with not having not having antibiotics available immediately”  30-year-old, female, Canada: “And sometimes the antibiotics are just based on their best guess right? When they give you the antibiotics, it's not be– at that time, They don't know what bacteria strain you have yet, so they will just give you what they think will work” |

*Note.* Code(s) from the Question Appraisal System – 99 [42]. Items are written as they are presented in the measure (i.e., with underlined or bold phrasing). Updated items were taken forward for testing in the second phase of patient cognitive interviews.

Hyphens (−) indicate that no changes were made to the corresponding item.

| Original instruction/item | Updated instruction/item | Code(s) | Quotation(s) |
| --- | --- | --- | --- |
| NEW SECTION | [New Section A –before social impact questions]  The following questions are about the impact of your UTI(s) on your personal wellbeing. | – | 24-year-old, female, UK: “Maybe some stuff [is missing] about how it’s impacted on your like psychological wellbeing or like mental health” |
|  | Thinking about how you have felt in the past TWO WEEKS, please indicate how strongly you agree or  disagree with the following statements: | – | – |
|  | Because of my UTI(s)… | – | – |
|  | A1. I have experienced feelings of anxiety. | – | 42-year-old, female, UK: “the psychological impact needs to be taken into account … it’s anxiety, concentration, mood, really”  71-year-old, female, USA: “I'm fine if I don't think about it. When I don't have an infection. But then all of a sudden, I'll get a twinge and I'll say, Oh, my God, and then it takes over. And I've heard this from other women, too. … But basically, I'm one of these people who sees both sides and accepts it, but when I feel an infection, it takes over my thinking, and I become, I would say borderline neurotic, like I can't take it anymore. I can't take it. I could go to sleep not wanting to wake up … Sometimes I just become so anxious until the symptoms can be alleviated by the pills” |
|  | A2. I have experienced feelings of low mood or depression. | – | 68-year-old, female, Australia: “You certainly haven’t talked about mood … I’m not prone to depression, but I would have to say, when I was really ill, I had very low mood … I just felt quite depressed about it”  26-year-old, female, Austria: “Stuff that hasn’t really gone into it is like, depression and stuff … UTIs and depression, they go hand in hand so often” |
|  | A3. I have felt hopeless about the future. | – | 53-year-old, female, UK: “There are fears about the future. So, when you talk about impact, one of my, the biggest impact for me right now is what does it mean for me as an old person? Because I’m 53 now and I will get old. And what does that mean for my care? What does it mean for my career? Because I have a very good employer right now, and that’s possibly not, you know, it’s not always going to be the case” |
|  | A4. I have had poor or disrupted sleep. | – | 68-year-old, female, Australia: “Sleep, you’ve haven’t asked. It’s dreadful, shocking impact on sleep … disrupted … big impact on sleep and lack of sleep can really knock you about”  42-year-old, female, UK: “Because sleep is obviously quite important to health, so then if you’ve got a chronic UTI, your sleep’s then disrupted, so then it because a vicious circle” |
|  | A5. I have been concerned about my diet. | – | 35-year-old, female, Netherlands: “Maybe diet … I don’t see it present there, and it affects a lot of people … I want to avoid certain foods because I fear they may contribute |
|  | A6. I have been unable to exercise as I normally would. | – | 26-year-old, female, Austria: “[Exercise] has definitely been impact … I feel like I can’t do anything that puts like pressure on my bladder … I did yoga and swimming especially, and I’m not really doing that anymore because I’m just afraid that it will come back” |
|  | Scale: 0 = strongly disagree; 10 = strongly agree | – | [to match other RUTIIQ subscales] |
| NEW INTRODUCTION | [At the very start of the questionnaire]  This questionnaire asks about the impact of your urinary tract infection (UTI) symptoms on your personal wellbeing, social activities, work and regular daily activities, and sexual activities. It also asks about your feelings of satisfaction with your UTI–related medical care. | – | [to match RUTISS]  56-year-old, female, UK: “[having an introduction to the domains at the top would be useful because] I could imagine ticking that and then coming on, if I come onto it later, I’ll be thinking, I’m gonna have to go back cos I’ve done that wrong now” |

*Note.* Code(s) from the Question Appraisal System – 99 [42]. Items are written as they are presented in the measure (i.e., with underlined or bold phrasing). Updated items were taken forward for testing in the second phase of patient cognitive interviews.

Hyphens (−) indicate that no changes were made to the corresponding item.

**Qualitative feedback and refinements made after the second phase of patient cognitive interviewing**

| Original instruction/item | Updated instruction/item | Code(s) | Quotation(s) |
| --- | --- | --- | --- |
| Introduction |  |  |  |
| This questionnaire asks about the impact of your urinary tract infection (UTI) symptoms on your personal wellbeing, social activities, work and regular daily activities, and sexual activities. It also asks about your feelings of satisfaction with your UTI–related medical care. | – | – | – |
| Section A |  | – | – |
| The following questions are about the impact of your UTI(s) on your personal wellbeing. | – | – | – |
| Thinking about how you have felt in the past TWO WEEKS, please indicate how strongly you agree or disagree with the following statements: | – | – | – |
| Because of my UTI(s)… | – | – | – |
| A1. I have experienced feelings of anxiety. | – | – | – |
| A2. I have experienced feelings of low mood or depression. | – | – | – |
| A3. I have felt hopeless about the future. | – | – | – |
| A4. I have had poor or disrupted sleep. | – | – | – |
| A5. I have been concerned about my diet. | I have been extra aware of what I eat and/or drink. | Knowledge: Attitude | 56-year-old, female, UK: “Just wondering what you're trying to capture about I have been concerned about my diet. I'm, presumably you're talking about nutrition levels, rather than the reduction of calories. I mean, I'm not concerned about either”  51-year-old, female, UK: “I think I'd prefer a question about has this impacted your diet? Or has this changed what you can eat? You know, I don't know. I just think, I prefer something about that rather than as it made you concerned about your diet”  42-year-old, female, Australia: “I would think if I have an onset of a UTI, I would think if I have, well, I have stopped drinking all wine and beer … it wouldn't be concerned, it would be more. I would be assessing what type of diet or alcohol intake I've take, you know, have had in the last … two weeks”  34-year-old, female, USA: “I always wonder what’s doing it or if it’s something I’m eating … I will always assess my diet” |
| A6. I have been unable to exercise as I normally would. | – | – | – |
| Scale: 0 = strongly disagree; 10 = strongly agree | – | – | – |

*Note.* Code(s) from the Question Appraisal System – 99 [42]. Items are written as they are presented in the measure (i.e., with underlined or bold phrasing). Updated items were taken forward for testing in the pilot test with patient participants.

Hyphens (−) indicate that no changes were made to the corresponding item.

| Original instruction/item | Updated instruction/item | Code(s) | Quotation(s) |
| --- | --- | --- | --- |
| Section B |  |  |  |
| The following questions are about the social impact of your UTI(s). | – | – | – |
| Thinking about how you have felt in the past TWO WEEKS, please indicate how strongly you agree or disagree with the following statements: | – | – | – |
| Because of my UTI(s)… | – | – | – |
| B1. I have felt that my close relationships have been impaired. | – | – | – |
| B2. I have felt that my ability to form new close relationships has been impaired. | – | – | – |
| B3. I have felt that my social activities have been impaired. | – | – | – |
| B4. I have felt alone or isolated from others. | – | – | – |
| B5. I have felt left out. | ITEM REMOVED | Clarity: Vague | 53-year-old, female, USA: “I think it would be, I don't feel left out. It's that I exclude myself. You know, thank you for inviting me, but I'm not going on that hike with you guys today”  51-year-old, female, Canada: “I would think of it as basically very similar to before. Isolated, alone, and you know, left out of being able to do things with other people and but I don't feel that way. Everybody else goes off and you know, has a picnic and I can't join kind of thing”  78-year-old, female, Canada: “No, it's not in me to feel left out or something. I couldn't care less. If I'm left out of something. I just go ahead and do it anyway, whatever. So it's just a personality thing I think” |
| B6. I have felt that I am no longer close to others. | – | – | – |
| B7. I have felt different from others. | – | – | – |
| B8. I have felt worried that I am a burden to others. | – | – | – |
| B9. I have avoided socialising more than I used to. | – | – | – |
| B10. I have felt embarrassed in social situations. | – | – | – |
| B11. I have felt anxious in social situations. | – | – | – |
| Scale: 0 = strongly disagree; 10 = strongly agree | – | – | – |

*Note.* Code(s) from the Question Appraisal System – 99 [42]. Items are written as they are presented in the measure (i.e., with underlined or bold phrasing). Updated items were taken forward for testing in the pilot test with patient participants.

Hyphens (−) indicate that no changes were made to the corresponding item.

| Original instruction/item | Updated instruction/item | Code(s) | Quotation(s) |
| --- | --- | --- | --- |
| Section C |  |  |  |
| The following questions are about the impact of your UTI(s) on your work. The term "work" is used here to include paid or unpaid employment, and/or regular daily activities, such as home management or caring responsibilities, studying, etc. | The following questions are about the impact of your UTI(s) on your work and/or regular daily activities. | Clarity: Vague | 42-year-old, female, Australia: “I wouldn't say work I would just say in your daily life. Because I mean, it looks, looks like you guys are covering everything from employment to just your daily home, you know, home management and caring responsibilities. So I would just say life, but yeah, it's very descriptive” |
| Thinking about how you have felt in the past TWO WEEKS, please indicate how strongly you agree or disagree with the following statements: | Thinking about how you have felt in the past TWO WEEKS, please indicate how strongly you agree or disagree with the following statements. Please consider the term “work” to include paid employment, volunteering, home management, caring responsibilities, and/or studying. | Clarity: Vague,  Technical terms | 51-year-old, female, Canada: “You might want to include volunteer in there, because to be honest, because I stopped working because of my cancer, so I don't work. And I don't know if unpaid employment is, is volunteer, but that's not clear to me … I’ve never actually heard [the term unpaid employment]”  71-year-old, female, USA: “Employment is never usually, here in the United States, ever used with unpaid employment … those two terms don’t seem to jive here … I would say paid work or … voluntary or unpaid activities” |
| Because of my UTI(s)… | – | – | – |
| C1. I have regularly missed full or partial days of work, home responsibilities, or studying. | – | – | – |
| C2. My ability to work was impaired. | – | – | – |
| C3. I have regularly put pressure on myself to work despite feeling unwell. | – | – | – |
| C4. The kind or amount of work I could do was limited. | – | – | – |
| C5. It was more difficult than usual to concentrate on my work. | – | – | – |
| C6. It was more difficult than usual to handle my workload. | – | – | – |
| C7. The quality of my work was lower than usual. | I have felt that the quality of my work was lower than usual. | Knowledge | 51-year-old, female, UK: “Now, in my head, the quality of my work is lower than usual, but actually, colleagues will tell me that it isn’t”  78-year-old, female, Canada: “Who’s judging the quality, anyway?” |
| C8. I experienced financial pressure (e.g. due to medical costs, missing work). | – | – | – |
| Scale: 0 = strongly disagree; 10 = strongly agree | – | – | – |

*Note.* Code(s) from the Question Appraisal System – 99 [42]. Items are written as they are presented in the measure (i.e., with underlined or bold phrasing). Updated items were taken forward for testing in the pilot test with patient participants.

Hyphens (−) indicate that no changes were made to the corresponding item.

| Original instruction/item | Updated instruction/item | Code(s) | Quotation(s) |
| --- | --- | --- | --- |
| Section D |  |  |  |
| The following questions are about the sexual impact of your UTI(s). | – | – | – |
| Do you feel your UTI(s) has/have impacted your sex life in the past two weeks? | – | – | – |
| Responses: Yes; No; Prefer not to say | – | – | – |
| If you selected “No” or “Prefer not to say”, please skip to section E. If you selected “Yes”, please continue with the rest of Section D. | – | – | – |
| Thinking about how you have felt in the past TWO WEEKS, please indicate how strongly you agree or disagree with the following statements: | – | – | – |
| D1. I have avoided sexual activity to minimise risk of developing or worsening of UTI symptoms. | – | – | – |
| D2. I have felt unable to enjoy sexual activity due to my UTI(s). | – | – | – |
| D3. I have been concerned about the impact of my UTI(s) on my sex life and/or sexual relationships. | – | – | – |
| D4. I have felt that my UTI(s) have made my sexual wellbeing worse. | – | – | – |
| Scale: 0 = strongly disagree; 10 = strongly agree | – | – | – |

*Note.* Code(s) from the Question Appraisal System – 99 [42]. Items are written as they are presented in the measure (i.e., with underlined or bold phrasing). Updated items were taken forward for testing in the pilot test with patient participants.

Hyphens (−) indicate that no changes were made to the corresponding item.

| Original instruction/item | Updated instruction/item | Code(s) | Quotation(s) |
| --- | --- | --- | --- |
| Section E |  |  |  |
| The following questions are about pain during or after sexual activity due to your UTI(s). Please consider both penetrative and non–penetrative sexual activity involving the genital and/or anal areas, with or without a partner(s). | – | – | – |
| Have you engaged in sexual activity in the past two weeks? | – | – | – |
| Responses: Yes; No; Prefer not to say | – | – | – |
| If you selected “No” or “Prefer not to say”, please skip to section F. If you selected “Yes”, please continue with the rest of Section E. | – | – | – |
| Thinking about how you have felt in the past TWO WEEKS, please indicate how strongly you agree or disagree with the following statements about your lower abdominal, genital and/or bladder pain or discomfort: | – | – | – |
| E1. I have experienced pain or discomfort here during sexual activity. | I have experienced pain or discomfort there during sexual activity. | Clarity: Vague | [Grammar] |
| E2. I have experienced pain or discomfort here within the 48 hours after sexual activity. | I have experienced pain or discomfort there within the 48 hours after sexual activity. | Clarity: Vague | [Grammar] |
| Scale: 0 = strongly disagree; 10 = strongly agree | – | – | – |
| If you experienced lower abdominal, genital and/or bladder pain or discomfort related to sexual activity in the past TWO WEEKS, please indicate how SEVERE it was: | – | – | – |
| E3. During sexual activity. | – | – | – |
| E4. Within the 48 hours after sexual activity. | – | – | – |
| Scale: 0 = not present; 1 = very mild; 10 = extremely severe | – | – | – |

*Note.* Code(s) from the Question Appraisal System – 99 [42]. Items are written as they are presented in the measure (i.e., with underlined or bold phrasing). Updated items were taken forward for testing in the pilot test with patient participants.

Hyphens (−) indicate that no changes were made to the corresponding item.

| Original instruction/item | Updated instruction/item | Code(s) | Quotation(s) |
| --- | --- | --- | --- |
| Section F |  |  |  |
| The following questions are about your feelings of satisfaction with your UTI–related medical care. | – | – | – |
| Thinking about how you have felt in the past TWO WEEKS, please indicate how strongly you agree or disagree with the following statements about your UTI–related medical care: | – | – | – |
| F1. I have generally felt content with the medical care I have been receiving. | – | – | – |
| F2. I have felt confident about being able to get the medical care I need. | – | – | – |
| F3. I have felt confident about being able to access UTI testing and treatment quickly enough. | – | – | – |
| F4. I have had easy access to the medical specialists I need. | – | – | – |
| F5. I have felt like my medical concerns are taken seriously. | – | – | – |
| F6. I have felt listened to by my healthcare provider(s). | – | – | – |
| F7. I have felt like my healthcare provider(s) treat me with respect and dignity. | – | – | – |
| F8. I have had confidence in the decisions made about my care and treatment. | – | – | – |
| F9. I have been as involved as I have wanted to be in the decisions made about my care and treatment. | – | – | – |
| F10. I have trusted my healthcare provider(s). | – | – | – |
| Scale: 0 = strongly disagree; 10 = strongly agree | – | – | – |

*Note.* Code(s) from the Question Appraisal System – 99 [42]. Items are written as they are presented in the measure (i.e., with underlined or bold phrasing). Updated items were taken forward for testing in the pilot test with patient participants.

Hyphens (−) indicate that no changes were made to the corresponding item.
