# Supplementary material for: Development and psychometric validation of a patient-reported outcome measure of recurrent urinary tract infection impact: the Recurrent UTI Impact Questionnaire
Source: Qual Life Res. 2023 Feb 6;32(6):1745–58. doi: 10.1007/s11136-023-03348-7 (PMC10172217; doi:10.1007/s11136-023-03348-7)
Supplement: Supplementary file 6 — Online Resource 6: Cognitive interview sampling and recruitment strategy (Stage III) (PDF 69 kb) [file 11136_2023_3348_MOESM6_ESM.pdf]

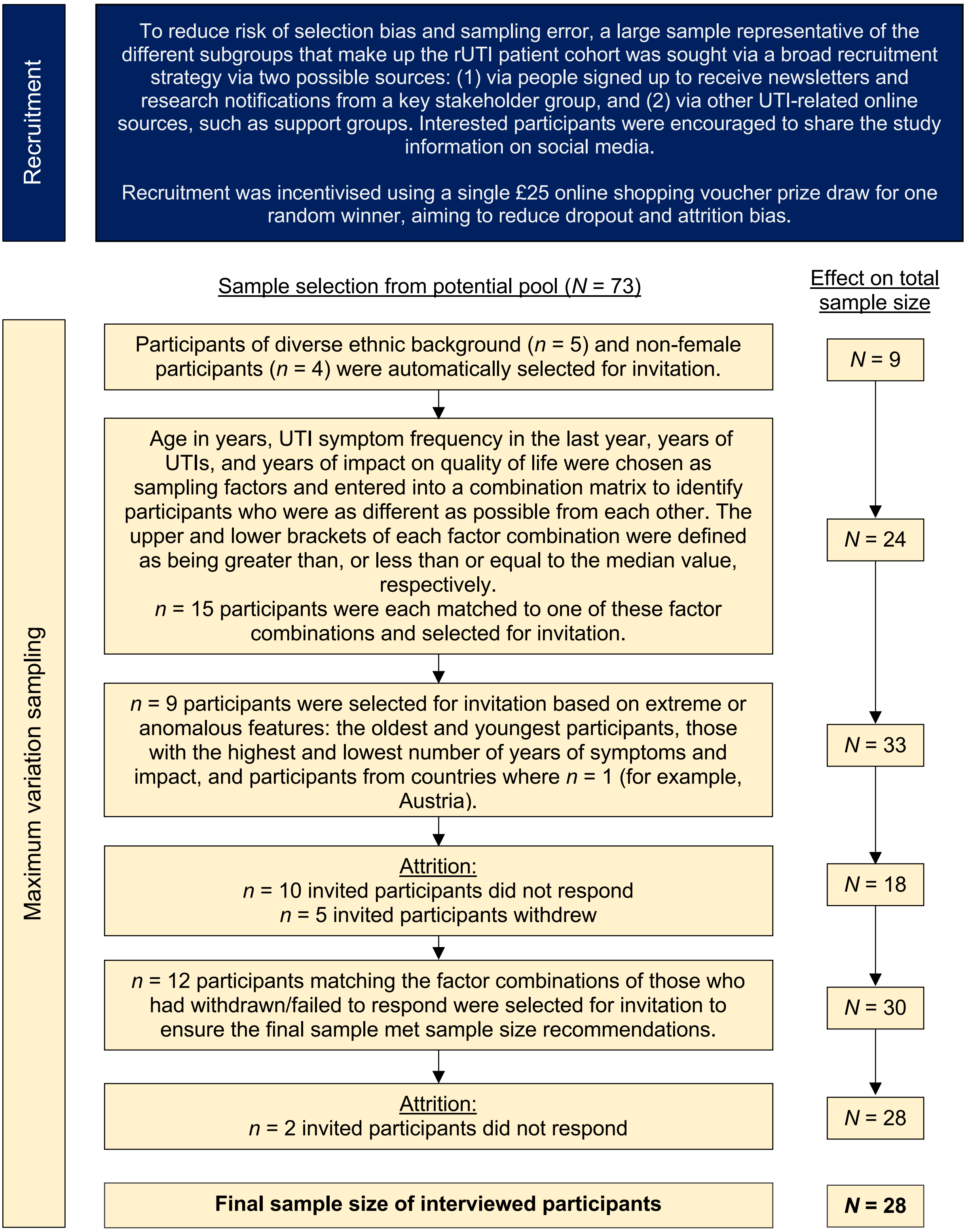

Note. A total of 73 potential participants completed the consent form and screening survey without being excluded. A maximum variation sampling strategy was applied to select as diverse a sample as possible from this potential pool, as described above [40]. This resulted in a final sample of 28 interviewed patient participants.
