# Supplementary material for: Development and psychometric validation of a patient-reported outcome measure of recurrent urinary tract infection impact: the Recurrent UTI Impact Questionnaire
Source: Qual Life Res. 2023 Feb 6;32(6):1745–58. doi: 10.1007/s11136-023-03348-7 (PMC10172217; doi:10.1007/s11136-023-03348-7)
Supplement: Supplementary file 7 — Online Resource 7: Cognitive interview topic guide (Stage III) (PDF 59 kb) [file 11136_2023_3348_MOESM7_ESM.pdf]

Topic Guide for Cognitive Interviews: Development of the Recurrent UTI Impact Questionnaire (RUTIIQ)

Use "think aloud" and "verbal probing" techniques following Willis' guidelines [36, 37].

1. **INTRO:**
   - What are your initial impressions of the questionnaire?

2. **SECTION A: SOCIAL IMPACT**

General probing questions:

- How easy or difficult is this to answer?
- What do you think the question is trying to find out?
- I noticed that you hesitated – what were you thinking about?
- How did you feel about the requirement to think about the impact of your UTI(s) over the past two weeks? / What do you think the best timeline would be for assessing the impact?
- How do you feel about the format of the questions?
- Is there anything you would change to make it better?

Possible question/section-specific probing questions:

- How did you work out how to use the scale of strongly disagree to strongly agree?
- **A1**: What does the phrase "form and maintain close relationships with others" mean to you?
- **A1** and **A2**: What does the word "impaired" mean to you?
- **A2**: What does the phrase "social activities" mean to you?
- What does the word "often" mean to you?
- **A3**: What does "isolated" mean to you?
- **A6**: What does the phrase "different from others" mean to you?
- **A7**: What does the word "burden" mean to you?
- **A10**: What does "used to" make you think about?
- Is there anything you feel is missing in terms of social impact due to UTIs?

3. **SECTION B: WORK IMPACT**

General probing questions:

- How easy or difficult is this to answer?
- What do you think the question is trying to find out?
- I noticed that you hesitated – what were you thinking about?
- How did you feel about the requirement to think about the impact of your UTI(s) over the past two weeks? / What do you think the best timeline would be for assessing the impact?
- How do you feel about the format of the questions?
- Is there anything you would change to make it better?

Possible question/section-specific probing questions:

- How clear is the definition of "work" here?
- To what extent does the definition of "work" given here apply to you?
- What does the word "often" mean to you?
- **B1**: What does the word "impaired" mean to you?
- **B3**: What does the word "limited" mean to you, as it is used in B3?
- **B5**: What does "handle your workload" mean to you?
- **B6**: How did you assess the quality of your work?
- **B7**: How did you work out the frequency with which you experienced financial pressure?
- **B7**: How easy was it to apply this question specifically to your experience of living with UTIs?

4. **SECTION C: SEXUAL PAIN**

General probing questions:

- How easy or difficult is this to answer?
- What do you think the question is trying to find out?
- I noticed that you hesitated – what were you thinking about?
- How do you feel about the format of the questions?
- Is there anything you would change to make it better?

Possible question/section-specific probing questions:

- How clear is the definition of "sexual activity" here?
- **Pre-screen question**: How easy or difficult did you find it to decide whether you were going to say "yes" or "no"? / What do you think influenced your answer?
- How do you interpret "sexual activity" as it is used here?
- How clear is the difference between **C1-2** and **C3-4**?
- Are there any aspects of the wording used in these questions that you feel should be adjusted? What would you adjust it to?
- How did you feel about the requirement to think about sexual activity in the past two weeks? / What do you think the best timeline would be for assessing the impact on sexual activity?

5. **SECTION D: SEXUAL IMPACT**

General probing questions:

- How easy or difficult is this to answer?
- What do you think the question is trying to find out?
- I noticed that you hesitated – what were you thinking about?
- How do you feel about the format of the questions?
- Is there anything you would change to make it better?

Possible question/section-specific probing questions:

- **Pre-screen question**: How easy or difficult did you find it to decide whether you were going to say "yes" or "no"? / What do you think influenced your answer?
- How did you work out how to use the scale of strongly disagree to strongly agree?
- How do you interpret "sexual activity" as it is used here?
- **D1**: What does the word "avoid" mean to you in this context?
- **D2**: What does the phrase "enjoy sexual activity" mean to you?
- **D3**: What types of impact do you think of in D3?
- **D4**: What does "sexual wellbeing" mean to you?
- How did you feel about the requirement to think about sexual impact in the past two weeks? / What do you think the best timeline would be for assessing the impact on sexual activity?

6. **SECTION E: PATIENT SATISFACTION**

General probing questions:

- How easy or difficult is this to answer?
- What do you think the question is trying to find out?
- I noticed that you hesitated – what were you thinking about?
- How did you feel about the requirement to think about the impact of your UTI(s) over the past two weeks? / What do you think the best timeline would be for assessing the impact?
- How do you feel about the format of the questions?
- Is there anything you would change to make it better?

Possible question/section-specific probing questions:

- How did you work out how to use the scale of strongly disagree to strongly agree?
- What type of medical care do you think about in these questions?
- **E1**: What does the word "content" mean to you in this context?
- **E4**: How do you assess whether or not your medical concerns are taken seriously?
- **E5**: How do you judge feeling "listened to"?
- **E7**: How do you assess how involved you have been in your medical care decisions?
- **E9**: What type of "medical specialists" do you think of in E9?

7. **OVERALL QUESTIONNAIRE**
   - What are your overall views on the questionnaire?
   - Is there anything you would change to make it better?
   - Is there anything else you'd like to say about this questionnaire that I haven't already asked?
   - How do you feel about the amount of time that these questions take to answer?
