# Supplementary material for: Development and psychometric validation of a patient-reported outcome measure of recurrent urinary tract infection impact: the Recurrent UTI Impact Questionnaire
Source: Qual Life Res. 2023 Feb 6;32(6):1745–58. doi: 10.1007/s11136-023-03348-7 (PMC10172217; doi:10.1007/s11136-023-03348-7)
Supplement: Supplementary file 8 — Online Resource 8: Pilot sampling and recruitment strategy (Stage IV) (PDF 94 kb) [file 11136_2023_3348_MOESM8_ESM.pdf]

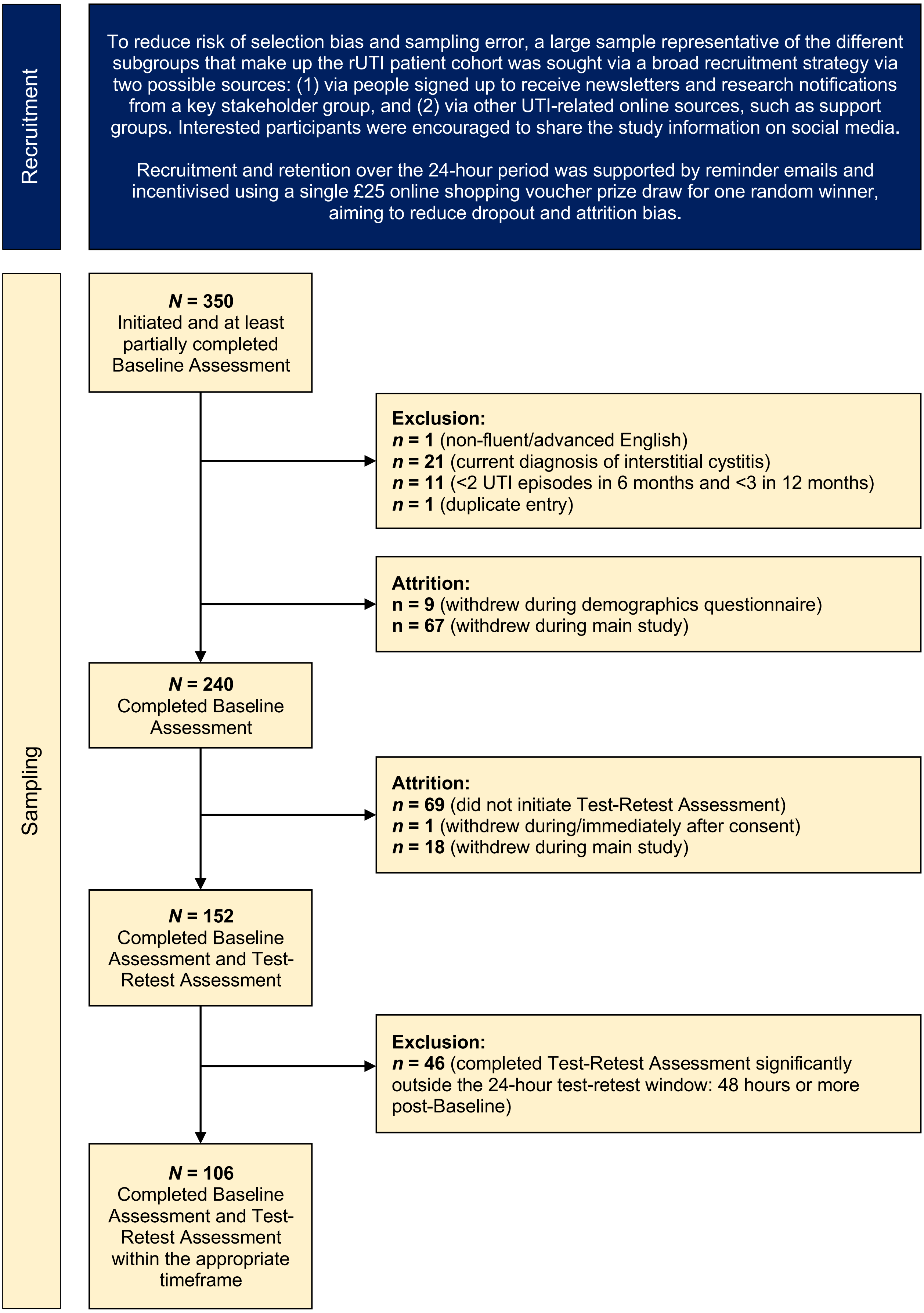

Note. Data from all 240 participants who completed the Baseline Assessment were included in every statistical test, except for the test-retest analyses which were conducted only with those who completed both the Baseline and Test-Retest Assessments (*N* = 106). Participants with missing data (for example, those who withdrew from the Baseline and/or Test-Retest Assessment without completion) were excluded from analysis.
