# Supplementary material for: Development and psychometric validation of a patient-reported outcome measure of recurrent urinary tract infection impact: the Recurrent UTI Impact Questionnaire
Source: Qual Life Res. 2023 Feb 6;32(6):1745–58. doi: 10.1007/s11136-023-03348-7 (PMC10172217; doi:10.1007/s11136-023-03348-7)
Supplement: Supplementary file 9 — Online Resource 9: Pilot data handling strategy (Stage IV) (PDF 39 kb) [file 11136_2023_3348_MOESM9_ESM.pdf]

Data preparation and assumption testing

1. Total scores were calculated for each of the Recurrent Urinary Tract Infection Impact Questionnaire (RUTIIQ) subscales at Baseline and Test-Retest Assessments. Total scores for the RUTIIQ as a whole measure were not computed to avoid misrepresenting multidimensional constructs as a single score [51].

2. Data collected using the following concurrent measures were scored according to their standard scoring systems: Patient Health Questionnaire 9 (PHQ-9) [45], Generalized Anxiety Disorder 7 (GAD-7) [46], University of Los Angeles Loneliness Scale Version 3 (UCLA-LS) [47], Work Productivity and Activity Impairment Questionnaire for Specific Health Problems (WPA:SHP) [48], Female Sexual Distress Scale – Revised [49], and Patient Satisfaction Questionnaire 18 (PSQ-18) [50].

3. The following assumptions were assessed:
    - Normality (Shapiro-Wilk p>.05 so non-parametric analyses were conducted where applicable)
    - Linearity (examination of scatter plots confirmed no violation)
    - Multicollinearity (Variance Inflation Factor scores for the target label data as well as possible demographic characteristics were sufficiently close to 1)
    - Independence of observations (Durbin-Watson statistic was approximately 2 for each subscale score)
    - Homoscedasticity (inspection of the standardised residuals plotted against standardised predicted values confirmed no violation)

4. Case-wise diagnostics of the subscale scores indicated five possible outliers outside 3*SD*; however, since these were not measurement errors and could feasibly reflect true variation within the population, they were not removed. Analyses conducted with and without these cases indicated that they had no statistically significant effect on the outcomes ($p > .05$).

Statistical analyses

Exploratory factor analysis (EFA):
- Suitability for EFA was checked through bivariate correlation matrices with coefficients greater than .80 examined for potential multicollinearity [52].
- Preliminary data analyses were undertaken to assess the factorability of the dataset using Bartlett's Test of Sphericity and the Kaiser-Meyer-Olkin Measure of Sampling Adequacy [53].
- Principal Axis Factoring was selected for application above Principal Components Analysis and Maximum Likelihood Analysis to facilitate determination of a latent factor structure [53, 54]. Varimax rotation with Kaiser Normalisation was applied to minimise cross-loading of items on factors [54].
- Visual inspection of scree plots determined the retained number of factors, combined with evaluation of eigenvalues exceeding Kaiser's criterion of 1 [55, 56].
- Items with extracted communalities less than .40 were removed, and a minimum factor loading of .40 was defined as acceptable a priori [54, 55].

Internal consistency was measured for each finalised post-EFA RUTIIQ subscale and the RUTIIQ as a whole measure using Cronbach's alpha.

To assess **test-retest reliability**, intraclass correlation coefficients (ICC) and their 95% confidence intervals were computed based on a single-rating, absolute-agreement, two-way mixed effects model, as recommended for test-retest reliability analysis of measures intended to demonstrate outcomes at an individual (single-rater) level [57].

Construct validity was assessed by computing the level of Spearman's correlation between each RUTIIQ subscale and the observed PHQ-9, GAD-7, UCLA-LS, WPAI:SHP, FSDS-R, and PSQ-18 scores.

Linear regression analyses were also conducted to examine whether there was a statistically significant effect of any demographic variables on the RUTIIQ scores (i.e., measurement invariance).

An **Automated Readability Index**, known to be especially useful when applied to technical, non-narrative text [48], was computed to estimate the literacy level required for comprehension of the RUTIIQ.
