# Supplementary material for: Development and psychometric validation of a patient-reported outcome measure of recurrent urinary tract infection impact: the Recurrent UTI Impact Questionnaire
Source: Qual Life Res. 2023 Feb 6;32(6):1745–58. doi: 10.1007/s11136-023-03348-7 (PMC10172217; doi:10.1007/s11136-023-03348-7)
Supplement: Supplementary file 10 — Online Resource 10: Recurrent Urinary Tract Infection Impact Questionnaire (RUTIIQ) (PDF 157 kb) [file 11136_2023_3348_MOESM10_ESM.pdf]

Recurrent Urinary Tract Infection Impact Questionnaire (RUTIIQ)

This questionnaire asks about the impact of your urinary tract infection (UTI) symptoms on your personal wellbeing, social activities, work and regular daily activities, and sexual activities. It also asks about your feelings of satisfaction with your UTI-related medical care.

A) The following questions are about the impact of your UTI(s) on your personal wellbeing.

Thinking about how you have felt in the past TWO WEEKS, please indicate how strongly you agree or disagree with the following statements:

	**Because of my UTI(s)…**	**0** Strongly disagree	**1**	**2**	**3**	**4**	**5**	**6**	**7**	**8**	**9**	**10** Strongly agree
A1.	I have experienced feelings of anxiety.	☐	☐	☐	☐	☐	☐	☐	☐	☐	☐	☐
A2.	I have experienced feelings of low mood or depression.	☐	☐	☐	☐	☐	☐	☐	☐	☐	☐	☐
A3.	I have felt hopeless about the future.	☐	☐	☐	☐	☐	☐	☐	☐	☐	☐	☐
A4.	I have had poor or disrupted sleep.	☐	☐	☐	☐	☐	☐	☐	☐	☐	☐	☐

B) The following questions are about the social impact of your UTI(s).

Thinking about how you have felt in the past TWO WEEKS, please indicate how strongly you agree or disagree with the following statements:

	**Because of my UTI(s)…**	**0** Strongly disagree	**1**	**2**	**3**	**4**	**5**	**6**	**7**	**8**	**9**	**10** Strongly agree
B1.	I have felt alone or isolated from others.	☐	☐	☐	☐	☐	☐	☐	☐	☐	☐	☐
B2.	I have avoided socialising more than I used to.	☐	☐	☐	☐	☐	☐	☐	☐	☐	☐	☐
B3.	I have felt embarrassed in social situations.	☐	☐	☐	☐	☐	☐	☐	☐	☐	☐	☐
B4.	I have felt that I am no longer close to anyone.	☐	☐	☐	☐	☐	☐	☐	☐	☐	☐	☐
B5.	I have felt anxious in social situations.	☐	☐	☐	☐	☐	☐	☐	☐	☐	☐	☐

C) The following questions are about the impact of your UTI(s) on your work and/or regular daily activities. Please consider the term "work" to include paid employment, volunteering, home management, caring responsibilities, and/or studying.

Thinking about how you have felt in the past TWO WEEKS, please indicate how strongly you agree or disagree with the following statements.

	**Because of my UTI(s)…**	**0** Strongly disagree	**1**	**2**	**3**	**4**	**5**	**6**	**7**	**8**	**9**	**10** Strongly agree
C1.	I regularly missed full or partial days of work, home responsibilities or studying.	☐	☐	☐	☐	☐	☐	☐	☐	☐	☐	☐
C2.	My ability to work was impaired.	☐	☐	☐	☐	☐	☐	☐	☐	☐	☐	☐
C3.	I regularly put pressure on myself to work despite feeling unwell.	☐	☐	☐	☐	☐	☐	☐	☐	☐	☐	☐
C4.	The kind or amount of work I could do was limited.	☐	☐	☐	☐	☐	☐	☐	☐	☐	☐	☐
C5.	It was more difficult than usual to concentrate on my work.	☐	☐	☐	☐	☐	☐	☐	☐	☐	☐	☐
C6.	I have felt that the quality of my work was lower than usual.	☐	☐	☐	☐	☐	☐	☐	☐	☐	☐	☐
C7.	It was more difficult than usual to handle my workload.	☐	☐	☐	☐	☐	☐	☐	☐	☐	☐	☐

D) The following questions are about the <u>sexual impact</u> of your UTI(s).

	Yes	No	Prefer not to say
Do you feel your UTI(s) has/have impacted your sex life in the past two weeks?	☐	☐	☐

If you selected "No" or "Prefer not to say", please skip to section E. If you selected "Yes", please continue with the rest of Section D.

Thinking about how you have felt in the past TWO WEEKS, please indicate how strongly you agree or disagree with the following statements:

		Strongly disagree 0	1	2	3	4	5	6	7	8	9	Strongly agree 10
D1.	I have avoided sexual activity to minimise risk of developing or worsening UTI symptoms.	☐	☐	☐	☐	☐	☐	☐	☐	☐	☐	☐
D2.	I have felt unable to enjoy sexual activity due to my UTI(s).	☐	☐	☐	☐	☐	☐	☐	☐	☐	☐	☐
D3.	I have been concerned about the impact of my UTI(s) on my sex life and/or sexual relationship(s).	☐	☐	☐	☐	☐	☐	☐	☐	☐	☐	☐
D4.	I have felt that my UTI(s) have made my sexual wellbeing worse.	☐	☐	☐	☐	☐	☐	☐	☐	☐	☐	☐

E) The following questions are about your feelings of satisfaction with your <u>UTI-related</u> medical care.

Thinking about how you have felt in the past TWO WEEKS, please indicate how strongly you agree or disagree with the following statements about your <u>UTI-related</u> medical care:

		Strongly disagree 0	1	2	3	4	5	6	7	8	9	Strongly agree 10
E1.	I have generally felt content with the medical care I have been receiving.	☐	☐	☐	☐	☐	☐	☐	☐	☐	☐	☐
E2.	I have felt confident about being able to get the medical care I need.	☐	☐	☐	☐	☐	☐	☐	☐	☐	☐	☐
E3.	I have felt like my medical concerns are taken seriously.	☐	☐	☐	☐	☐	☐	☐	☐	☐	☐	☐
E4.	I have had confidence in the decisions made about my care and treatment.	☐	☐	☐	☐	☐	☐	☐	☐	☐	☐	☐
E5.	I have felt confident about being able to access UTI testing and treatment quickly enough.	☐	☐	☐	☐	☐	☐	☐	☐	☐	☐	☐
E6.	I have felt listened to by my healthcare provider(s).	☐	☐	☐	☐	☐	☐	☐	☐	☐	☐	☐
E7.	I have had easy access to the medical specialists I need.	☐	☐	☐	☐	☐	☐	☐	☐	☐	☐	☐
E8.	I have felt like my healthcare provider(s) treat me with respect and dignity.	☐	☐	☐	☐	☐	☐	☐	☐	☐	☐	☐
E9.	I have been as involved as I have wanted to be in the decisions made about my care and treatment.	☐	☐	☐	☐	☐	☐	☐	☐	☐	☐	☐
E10.	I have trusted my healthcare provider(s).	☐	☐	☐	☐	☐	☐	☐	☐	☐	☐	☐

Thank you. This is the end of the questionnaire.
