# Supplementary material for: Development and psychometric validation of a patient-reported outcome measure of recurrent urinary tract infection impact: the Recurrent UTI Impact Questionnaire
Source: Qual Life Res. 2023 Feb 6;32(6):1745–58. doi: 10.1007/s11136-023-03348-7 (PMC10172217; doi:10.1007/s11136-023-03348-7)
Supplement: Supplementary file 11 — Online Resource 11: Pilot descriptive statistics (Stage IV) (DOCX 15 kb) [file 11136_2023_3348_MOESM11_ESM.docx]

**Online Resource 11.** Descriptive statistics for all instruments measured in the pilot study.

| Measure (max. score) | *n* | *M* | *SD* | Range |
| --- | --- | --- | --- | --- |
| RUTIIQ |  |  |  |  |
| Personal wellbeing (40) | 240 | 26.2 | 11.1 | 0–40 |
| Social wellbeing (50) | 240 | 23.3 | 15.9 | 0–50 |
| Work/activity interference (70) | 240 | 35.8 | 23.1 | 0–70 |
| Sexual wellbeing (40) | 183 | 36.2 | 6.3 | 5–40 |
| Patient satisfaction (100) | 240 | 36.6 | 28.5 | 0–100 |
| PHQ-9 (27) | 240 | 11.5 | 7.22 | 0–27 |
| GAD-7 (21) | 240 | 9.23 | 6.21 | 0–21 |
| UCLA-LS (80) | 240 | 48.8 | 13.9 | 21–79 |
| WPAI-SHP |  |  |  |  |
| % Work impairment (100) | 240 | 44.5 | 33.3 | 0–100 |
| % Activity impairment (100) | 240 | 45.7 | 29.6 | 0–100 |
| FSDS-R (52) | 197 | 30.3 | 13.7 | 0–52 |
| PSQ-18 (average) | 240 | 17.9 | 5.32 | 7.25–31.75 |

*Note.* *M* = mean. *SD* = standard deviation. RUTIIQ = Recurrent Urinary Tract Infection Impact Questionnaire. PHQ-9 = Patient Health Questionnaire 9 [45]. GAD-7 = Generalized Anxiety Disorder 7 [46]. UCLA-LS = University of Los Angeles Loneliness Scale Version 3 [47]. WPAI:SHP = Work Productivity and Activity Impairment Questionnaire for Specific Health Problems [48]. FSDS-R = Female Sexual Distress Scale – Revised [49]. PSQ-18 = Patient Satisfaction Questionnaire 18 [50]

*N* = 240 for computation of descriptive statistics, Cronbach’s alpha, and Spearman’s ρ for all subscales except for the *sexual wellbeing* subscale due to non-compulsory questions (*N* = 183).
