# Supplementary material for: Development and psychometric validation of a patient-reported outcome measure of recurrent urinary tract infection impact: the Recurrent UTI Impact Questionnaire
Source: Qual Life Res. 2023 Feb 6;32(6):1745–58. doi: 10.1007/s11136-023-03348-7 (PMC10172217; doi:10.1007/s11136-023-03348-7)
Supplement: Supplementary file 12 — Online Resource 12: Pilot analysis - age (Stage IV) (DOCX 15 kb) [file 11136_2023_3348_MOESM12_ESM.docx]

### **Online Resource 12.** Linear regression results for age as a predictor of RUTIIQ scores.

| Subscale | *R^2^_Adj_* | *F* | *df* | β | 95% CI | |
| --- | --- | --- | --- | --- | --- | --- |
|  |  |  |  |  | LB | UB |
| Personal wellbeing | .07*** | 18.6 | 1, 238 | -.17 | -.25 | -.09 |
| Social wellbeing | .04** | 10.9 | 1, 238 | -.19 | -.31 | -.08 |
| Work/activity interference | .05*** | 14.0 | 1, 138 | -.31 | -.48 | -.15 |
| Sexual wellbeing | -.006 | .00 | 1, 181 | .00 | -.06 | .06 |
| Patient satisfaction ^a^ | .07*** | 18.3 | 1, 238 | -.44 | -.24 | -.64 |

*Note.* *N* = 240 (except for sexual wellbeing: *N* = 183). Greater scores indicate greater impact. Mean age = 45.0 (*SD* = 17.3). *df* = degrees of freedom. CI = confidence interval. LB = lower bound; UB = upper bound.

***p* < .01. ****p* < .001.

^a^ Reverse-scored.
