# Supplementary material for: Development and psychometric validation of a patient-reported outcome measure of recurrent urinary tract infection impact: the Recurrent UTI Impact Questionnaire
Source: Qual Life Res. 2023 Feb 6;32(6):1745–58. doi: 10.1007/s11136-023-03348-7 (PMC10172217; doi:10.1007/s11136-023-03348-7)
Supplement: Supplementary file 13 — Online Resource 13: Pilot analysis - gender (Stage IV) (DOCX 15 kb) [file 11136_2023_3348_MOESM13_ESM.docx]

### **Online Resource 13.** Gender differences in RUTIIQ scores (ANOVA results).

| Subscale | *R^2^_Adj_* | *F* | *df* | 95% CI | | Mean difference | | |
| --- | --- | --- | --- | --- | --- | --- | --- | --- |
|  |  |  |  | LB | UB | Fem–Male | NB–Fem | NB–Male |
| Personal wellbeing | .04* | 4.32 | 2 | -.25 | -.09 | 13.5* | 8.50 | -5.00 |
| Social wellbeing | .04* | 4.59 | 2 | -.31 | -.08 | 18.8 | 21.3 | 2.50 |
| Work/activity interference | .05* | 5.47 | 2 | -.48 | -.15 | 27.0* | 34.7 | 7.75 |
| Sexual wellbeing | .04* | 4.83 | 2 | -.06 | .06 | 9.70* | .45 | 9.25 |
| Patient satisfaction ^a^ | .00 | 1.16 | 2 | -.24 | -.64 | 20.7 | .24 | -20.5 |

*Note.* *N* = 240 (except for sexual wellbeing: *N* = 183). *df* = degrees of freedom. CI = confidence interval. LB = lower bound; UB = upper bound. Fem = female; NB = non-binary.

Bonferroni post-hoc analysis of a one-way ANOVA indicated that significant gender differences in this sample laid between female and male participants (not non-binary participants).

**p* < .05.

^a^ Reverse-scored.
